# Exploring the Applications of Artificial Intelligence in Dental Image Detection: A Systematic Review

**DOI:** 10.3390/diagnostics14212442

**Published:** 2024-10-31

**Authors:** Shuaa S. Alharbi, Haifa F. Alhasson

**Affiliations:** Department of Information Technology, College of Computer, Qassim University, Buraydah 52571, Saudi Arabia; hhson@qu.edu.sa

**Keywords:** artificial intelligent, diagnostic imaging, diagnosis, deep learning, deep neural networks, machine learning, medical image processing, systematic review

## Abstract

Background: Dental care has been transformed by neural networks, introducing advanced methods for improving patient outcomes. By leveraging technological innovation, dental informatics aims to enhance treatment and diagnostic processes. Early diagnosis of dental problems is crucial, as it can substantially reduce dental disease incidence by ensuring timely and appropriate treatment. The use of artificial intelligence (AI) within dental informatics is a pivotal tool that has applications across all dental specialties. This systematic literature review aims to comprehensively summarize existing research on AI implementation in dentistry. It explores various techniques used for detecting oral features such as teeth, fillings, caries, prostheses, crowns, implants, and endodontic treatments. AI plays a vital role in the diagnosis of dental diseases by enabling precise and quick identification of issues that may be difficult to detect through traditional methods. Its ability to analyze large volumes of data enhances diagnostic accuracy and efficiency, leading to better patient outcomes. Methods: An extensive search was conducted across a number of databases, including Science Direct, PubMed (MEDLINE), arXiv.org, MDPI, Nature, Web of Science, Google Scholar, Scopus, and Wiley Online Library. Results: The studies included in this review employed a wide range of neural networks, showcasing their versatility in detecting the dental categories mentioned above. Additionally, the use of diverse datasets underscores the adaptability of these AI models to different clinical scenarios. This study highlights the compatibility, robustness, and heterogeneity among the reviewed studies. This indicates that AI technologies can be effectively integrated into current dental practices. The review also discusses potential challenges and future directions for AI in dentistry. It emphasizes the need for further research to optimize these technologies for broader clinical applications. Conclusions: By providing a detailed overview of AI’s role in dentistry, this review aims to inform practitioners and researchers about the current capabilities and future potential of AI-driven dental care, ultimately contributing to improved patient outcomes and more efficient dental practices.

## 1. Introduction

Over the last few decades, medical imaging methods such as Computerized Tomography (CT) and X-rays have been used to identify, detect, and treat many illnesses. Moreover, there are various methods for developing rapid diagnosis equipment for dental caries, such as assessing commonly used machine learning approaches on the impacts of annual parenteral examinations, and the use of classification techniques employing two distinct phases: digital image processing and characterization.

From the 1970s to the 1990s, clinical image recognition was initially performed by sequential-based low-level raster production (edge and line spectrometer filters, morphological operation) and numerical methods (appropriate lines, groups, and elliptical) to begin building rule-based mechanisms that solved specific tasks [1,2]. Dental informatics is a new and developing topic in dentistry with the potential to enhance treatment and diagnostics, save time, and lessen stress and exhaustion in daily practice [3,4]. In general, and in dentistry in particular, a variety of types of data are generated, including high-resolution radiography, continuously monitoring biosensors, and electronic records [5]. Computer applications can assist dental professionals in making decisions regarding, among other things, protection, diagnostics, and treatment planning [6].

In a prior Korean survey, only 21% of individuals visited dental centers and hospitals for dental care and examinations [7]. Therefore, the frequency may be much lower in low- and intermediate-income societies where dental inspections are costly and not reimbursed by insurance. Therefore, advanced screening systems that most of the public can conveniently use will help boost the number of dental caries assessments.

Artificial Intelligence (AI) has profoundly advanced the field of dentistry, integrating seamlessly into clinical workflows. It has transformed dentistry by enhancing diagnostic imaging, treatment planning, patient management, and workflow optimization. It improves image analysis, automates charting, and predicts treatment outcomes. It enhances diagnostic imaging through sophisticated algorithms that improve the accuracy of radiographic and CT scan analysis, identifying pathologies such as caries and bone resorption with precision. In treatment planning, AI employs predictive analytics for personalized therapeutic strategies and optimizes orthodontic device fabrication, such as aligners. Furthermore, AI provides real-time clinical decision support and comprehensive risk assessments, improving patient outcomes. It also contributes to professional education through advanced simulation training. Also robotic surgery could used to assist in precise surgical procedures, enhancing accuracy and reducing recovery time. Despite challenges like integration and data privacy, AI significantly boosts efficiency and accuracy in dental practices. Deep learning (DL) has been demonstrated to work well in image-based diagnostics across various disciplines [8]. Convolutional neural networks (CNNs) are a popular option for interpreting medical images in DL applications, which have progressed incredibly quickly over the past decade [9]. In medicine, CNNs have been successfully used to detect skin cancer during skin screenings, diabetic retinopathy during eye examinations, and breast cancer during mammograms [10].

CNNs have lately been used in dentistry to identify apical lesions, caries on bitewing radiographs, and periodontal bone loss, as well as to classify medical images [11,12]. These types of Artificial Neural Networks (ANNs) can be used to segment and classify structures, such as teeth or cavities, as well as to detect them [13]. An image database is required for the training and optimization of ANNs.

This study rationally focused on reviewing the current state of Artificial Intelligence (AI) in dentistry and state-of-the-art applications, including the recognition of teeth cavities, filled teeth, crown predictions, oral surgery, and endodontic therapy.

The purpose of this systematic review is to understand and compare the current applications of machine learning in the care of dental patients. This will enable us to assess their diagnostic and prognostic accuracy. As part of the study, we will identify areas of development for ML applications in the dental care field. In addition, we will suggest improvements to research methodology that will facilitate the implementation of ML technologies in services and improve clinical treatment guidelines based on the results of future studies.

## 2. Materials and Methods

This review was conducted in accordance with PRISMA guidelines [14] for preferred reporting items for systematic reviews and meta-analyses of diagnostic test accuracy studies.

### 2.1. Research Questions

1. How\Which the ML\DL Technique can be used to built an efficient dentistry diagnostic support system?
2. What are the possible optimizition techniques used by different methods to improve their performance?
3. What are each optimal methods for each teeth target?
4. What is the future of clinical applications in dentistry filed?

### 2.2. Data Source

To ensure a comprehensive and relevant collection of data for this systematic review, an extensive search of electronic databases was performed. The selection criteria were carefully designed to capture the forefront of research in artificial intelligence applications within dentistry. This search targeted major databases recognized for their rich accumulation of peer-reviewed articles, including Science Direct, PubMed (MEDLINE), arXiv.org, MDPI, Nature, Google Scholar, Scopus, and Wiley Online Library. The period from January 2013 to February 2024 was covered to include the most recent advances. Keywords were meticulously chosen to reflect critical areas in dental AI research, such as ‘teeth segmentation’, ‘detection of dental caries’, and ‘computer-aided diagnosis’, among others. This strategy was aimed not only at harnessing the most pertinent studies but also at ensuring that the scope of findings remained tightly aligned with the evolving landscape of AI in dental practice. Table 1 below summarizes the databases, time range, and specific keywords that framed our research strategy.

### 2.3. Resources Selection

**Figure 1:**
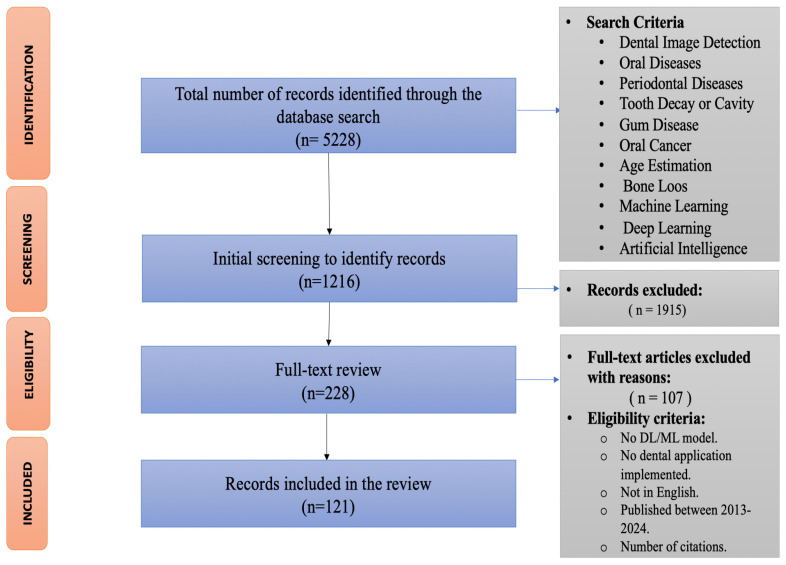
Detailed flowchart of study selection.

Full-length articles were retrieved from the journals. As part of the screening process, the two authors organize a focus group in order to ensure that the eligibility criteria and inclusion criteria are met. A list of the titles, authors, dates of publication, places of publication, and full abstracts of the literature obtained through the above-mentioned search protocol was imported into Microsoft Excel 2023. Using the software, duplicates were removed from the list of literature and the remaining article abstracts were screened using eligibility criteria. The required articles for this review study were selected in two stages. The first stage was the selection of articles based on the title and abstracts related to our research topic. The preliminary search yielded 5228 articles that were appropriate to address the study’s aim, then due to duplication, 4012 articles were removed. Hence, the two authors retrieved 1216 articles at the second stage of selection. In the next stage, they followed a criterion to include research papers. For the purposes of the review, all authors were satisfied with the exclusion and inclusion of papers. In order to avoid missing relevant literature, criteria were devised after a focus group consisting of the two authors above reviewed preliminary papers. Figure 1 shows the detailed flowchart of our study selection based on PRISMA-DTA methodology.

### 2.4. Inclusion and Exclusion Criteria

- The article must be focused on AI, and its application should be one of the related assigned dentistry applications and including the statistical analysis for the results.
- The article must include reference to or creation of datasets that are used to assess a model.

This criterion reduced the number of articles to (121). All the articles were read completely.

### 2.5. Performance and Accuracy Measures

Our study of the evolution of AI trends in dentistry over the years was based on the developments contained in these articles. As a general rule, the following performance evaluation metrics are most frequently used in the classification, segmentation, and detection of teeth problems: Accuracy, Precision, Sensitivity, Specificity, F1-score, Jaccard index, MAE, RMSE, R2, MRE and SDR. Table 2 summarize the statistical performance indicators used in the analyzed papers.

Due to the inclusion of accuracy terms in the search criteria, no papers were excluded for containing accuracy measurements not specified in the search criteria.

### 2.6. Data Synthesis and Analysis

Main characteristics of included caries and teeth targeted studies were used to group the extracted data according to its depth. They were also grouped based on their validation metrics used and their values that allowed direct comparison of data between studies. As part of the study, all outcome measures were extracted and analyzed in a standard format, including a complete definition of accuracy regardless of the measure used by the included papers to document this. In addition, each study included was evaluated based on QUADAS-2 quality assessment [15]. More details can be found later in Section 3.1.

## 3. Results

**Figure 2:**
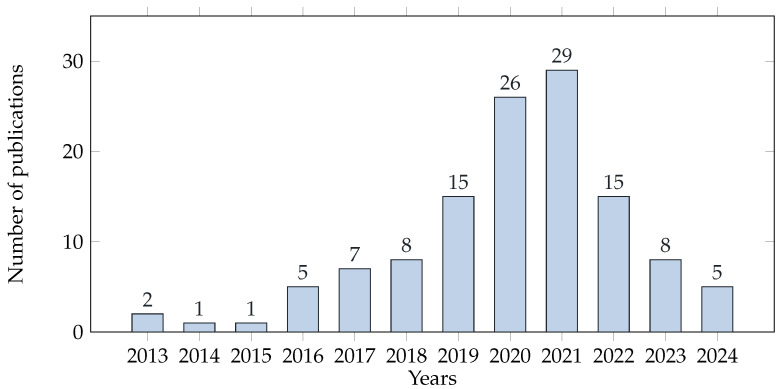
Artificial intelligence in dentistry research trends.

In total, 5228 papers were identified in this review paper. After eliminating duplicate titles, we were left with 1216, which were then evaluated for abstracts and excluded based on exclusion criteria (i.e., ref. [16] is excluded because no DL or ML model applied). The remaining articles (n = 228) were reviewed in their full-text forms. Based on the eligibility criteria displayed in Table 1, 121 studies were selected with multiple forms of machine learning. The included papers have been conducted over the past decade (between 2013–2024) as illustrated in Figure 2.

**Figure 3:**
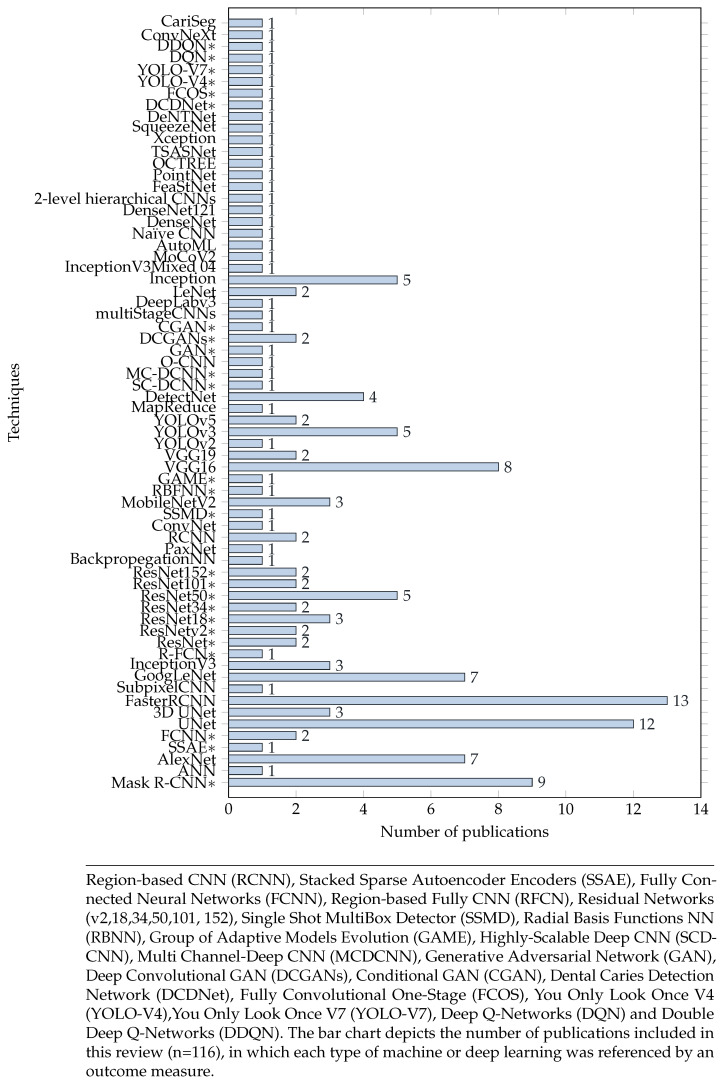
Graphical display of machine and deep learning models in included studies, where (*) indicates the full name of the model.

As the study contains many studies with a variety of characteristics and demographics. Table 3 and Table 4 provide a comprehensive comparison of study characteristics, Section 4 provide a details description of the included studies. All the papers included in this review were published between 2013 and 2022 and used a different set of data radiography listed before in Table 5. There was a wide variation in the Machine Learning algorithms have been applied across studies. The majority of studies used convolutional neural networks (CNN), U-nets, or R-CNNs. As display in Figure 3, around 60% of the studies used CNNs, including their two extensions, U-net (n = 12) or 3D U-net (n = 3) and faster R-CNN (n = 13) or mask R-CNN (n = 9).

### 3.1. Risks of Bias Assessment

**Figure 4:**
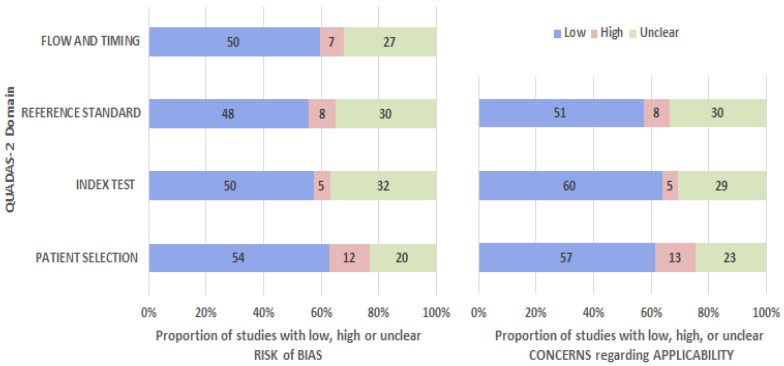
QUADAS-2 quality assessment graphs depict individual bias risk and concerns regarding applicability.

Throughout all of the studies, AI has been assessed for its diagnostic accuracy in a variety of specific areas of dentistry. QUADAS-2, a commonly used tool in the literature for risk of bias assessment, was used to assess the risk of bias [15]. There was a high level of risk associated with the studies conducted on humans in order to establish the reference standard. There were 7% of studies in the present analysis that reported a high risk of bias for the reference standard. Approximately 7% of the studies in the present analysis reported a high risk of bias regarding the reference standard. As AI technology relies on standardized data feeds, AI had little impact on final output flow or timeframe and was thus classified as a low-risk technology. The current systematic review reported a low risk of bias in the index test and in flow and timing (50%). However, the applicability arm of QUADAS-2 provided comparable results, as shown in Figure 4.

There is a great deal of interest in the topic of teeth caries as shown in Table 3. In some approaches, caries were detected in a large or small dataset, while in others, caries depth was used to determine treatment protocols.

The most notable growth in dental segmentation and classification, as shown in Table 4 can be summarized in two points:

1. In the segmentation domain, graph-based CNN overcomes many other segmentation methods due to the graph’s ability to avoid ambiguous labeling of other teeth [17]. Some approaches yielded good accuracy in detecting the 3D dental model using the 3D CNN model based on hierarchical voxel OCTREE and conditional random field CRF model [18].
2. In the classification domain, several studies focused on classifying the teeth, such as [19,20,21]. Some studies used the same models to detect the problems that affect the teeth [22] or their condition [23].

The most widely used network to enhance outcomes of teeth detection and teeth numbering is faster R-CNN because of its algorithm for selectively generating search region proposals.

Assessment measurement are varieties among included studies. Summarize of these assessment measurement describe in Table 2. According to Table 3 and Table 4, there were 11 out of 29 using Accuracy as assessment measurement. To this end, it is important to note that [24] and have unclear information about the value of accuracy test for their approach.

**Figure 5:**
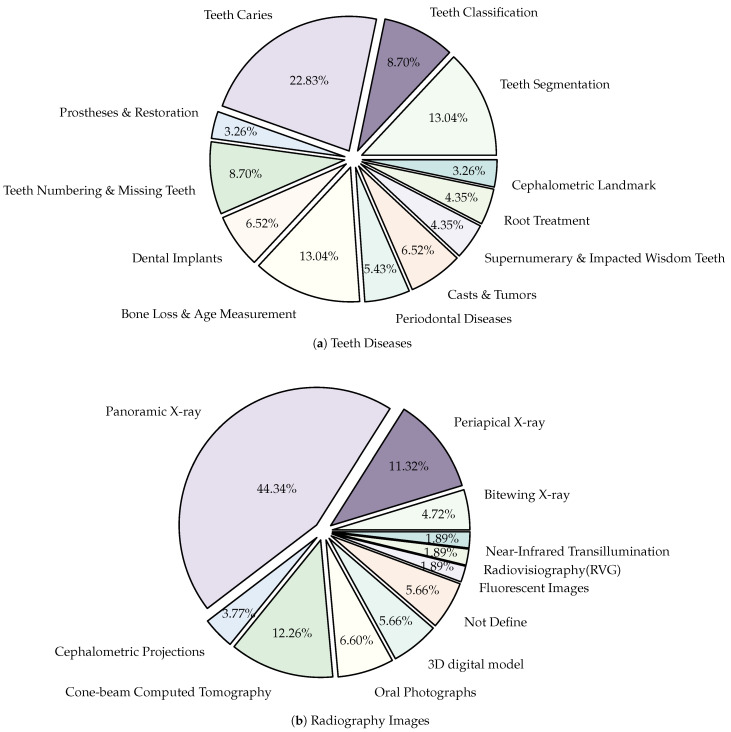
The focus distribution of dental detection: (**a**) Percentage of research published based on the types of teeth diseases, (**b**) Percentage of research published based on the types of radiography images.

A shown in Figure 5b, panoramic X-ray images are the most popular radiographic method used in the literature [13,25]. In panoramic dental X-rays, a relatively modest dosage of ionizing radiation is used to produce an image that includes the whole mouth. Therefore, this type of image is more suitable in diagnoses of teeth diseases, in order to plan root treatment [26,27], in diagnosis of gum [28,29] and jaw bone [30] diseases. In addition, it is frequently used by dentists and oral surgeons in routine practice or for non-medical purposes such as age estimation [24] or for preprocessing tasks such as teeth numbering [4], classification [31] and segmentation [32]. The techniques of NN and AI can be applied to a variety of radiological studies, such as the periapical X-ray and the CBCT. However, there is a shortage of data availability for both periapical X-rays and CBCT. It is worth to mention missing information regarding the dataset. Some methods [33,34] have missing data such as radiography type and number of images. Others such as [21] has missing number of images used in there method.

## 4. Machine Learning/Deep Learning for Dental Disease Detection

Currently, there is a growing interest in applying Artificial Intelligent (AI) strategies and image processing for medical image classification, detection, segmentation, and analysis. Generally, many dental applications and different modalities are used in dental imaging [13]. Some researchers design applications for specific types of dental diseases, while others focus on distinguishing and recognizing different variables, such as distinguishing the teeth from other tissues.

### 4.1. Caries Targeted Studies

Early detection of dental caries (a.k.a cavity) can prevent tooth damage and save expensive healthcare costs. Thus, an effective modality for the early detection of dental caries is a crucial subject in dental research [35]. From 2015 to 2024, twenty four studies were conducted on dental caries. The details of these studies can be found in Supplementary File Section (S1.1).

Table 3 summarizes the main characteristics and outcomes that were measured of included (caries) targeted studies.

**Table 3:** Main characteristics of included caries targeted studies.

Author	Year	Journal Rank (SJR)/ Conference Rank (Qualis)	Radiography	# of Images	ML/DL Model	Validation Metrics	Values
Ali et al. [33]	2016	B3	–	–	Stacked Sparse Autoencoder Encoders (SSAE)	AUC ROC	97%
Prajapati et al. [36]	2017	Not Yet Assigned	Radiovisiogra-phy image	251	–	Accuracy	0.875
Srivastava et al. [37]	2017	ArXiv	Bitewing	3000	FCNN (deep fully CNN)	Recall, Precision, F1-Score	0.805, 0.615, 0.7
Hatvani et al. [38]	2018	Q1	CBCT	5680 cross-sectional and 1824 slices	U-net & Subpixel CNN	Peak Signal-to-Noise Ratio(PSNR) Similarity index	0.9101
Lee et al. [39]	2018	Q1	Periapical image	3000	GoogLeNet Inception v3	Accuracy, AUC	premolar, molar, and both premolar and molar: 0.89, 0.88, 0.82, 0.917, 0.89, 0.845
Zhang et al. [40]	2018	Q1	Periapical	700	Faster-R-CNN, region-based fully convolutional networks (R-FCN)	Precision, Recall	0.958, 0.961
Casalengo et al. [41]	2019	Q1	Near-infrared transillumination	217	U-net	AUC	0.836 (occlusal lesion) and 0.856 (proximal lesion)
Schwendicke et al. [10]	2019	Q1	Near-infrared light transillumination	226	ResNet18 ResNet50	AUC, Sensitivity, Specificity and Positive, Negative predictive Values (PPV/NPV)	0.74, 0.59, 0.76
Geetha et al. [42]	2020	Not Yet Assigned	Radiovisiography image	105	Back-propagation NN	Accuracy, Precision, Recall	0.971, 0.987
Haghanifar et al. [43]	2020	ArXiv	Panoramic X-rays	470	PaXNet	Accuracy, Recall	86.05%, 69.44%, 90.52%
Lee et al. [44]	2020	Q2	Panoramic X-rays	846	R-CNN	F1 score, precision, Recall, mean Intersection over Union (IoU)	0.875, 0.858, 0.893, 0.877
Sonavane et al. [45]	2021	Not Yet Assigned	Oral photographs	74	Sequential model	Accuracy	71.43%
Sonavane et al. [45]	2021	Q1	Bitewing	304	U-Net	Precision, Recall, F1-score	63.29%, 65.02%, 64.14%
Bui et al. [46]	2021	Q2	Panoramic X-rays	533	Fusion feature and deep activated	Accuracy, Sensitivity, Specificity	91.70%, 90.43%, 92.67%
Ding et al. [47]	2021	Q3	Oral photographs	3990	YOLOv3	mAP, Precision, Recall, F1-score, AP	56.20%, 76.92%, 49.59%, 55.63%
Zheng et al. [48]	2021	Q3	Panoramic X-rays	844	VGG19, Inception V3, ResNet18	Accuracy, Precision, Sensitivity, Specificity	0.82, 0.81, 0.85, 0.82
Cantu et al. [49]	2020	Q1	Bitewing	3686	U-Net	Intersection-over-Union (IoU)	0.80
Zhang et al. in [50]	2022	Q1	Oral photographs	3932	ConvNet, Single Shot MultiBox Detector	AUC, Confidence interval	85.65% (95%, 82.48% to 88.71%).
kuhnisch et al. [51]	2022	Q1	Oral photographs	2417	MobileNet- V2	Sensitivity, Specificity and AUC	89.6%, 94.3%, 0.964
Day et al. [52]	2023	Q2	Panoramic X-rays	746 occlusal, 1627 proximal and 378 cervical caries	DCDNet	F-score, mIoU and Accuracy	97.79% 93.64%, 93.61%
Esmaeilyfard et al. [53]	2024	Q1	CBCT	382 (with caries) and 403 (noncarious)	Multiple-input CNN	Accuracy, Sensitivity, Specificity and F-score	95.3%, 92.1%, 96.3%, 93.2%
Chaves et al. [54]	2024	Q1	Bitewing X-ray	425	Mask-RCNN	ROC, Sensitivity, Specificity and F-score	0.806, 0.804, 0.689, 0.719

### 4.2. Teeth Targeted Studies

#### 4.2.1. Teeth Segmentation

Teeth detection has been a research subject for at least the last two decades, mainly relying on threshold and region-based, and machine learning methods [55]. This paper explores the progress made through machine/deep learning methods in segmenting teeth. The segmentation of teeth from different radiography images has been investigated in sixteen studies. Supplementary File Section (S1.2.1) contains details of these studies.

#### 4.2.2. Tooth Classification

This section contains the tooth classification methods that classify the type of teeth, the problem affecting the teeth, or the condition. Other classification studies focusing on solving other dental fields are distributed in other sections. The classification of tooth types was carried out in seven studies between 2012 and 2024. Where tow study proposed to classified different teeth problems. In addition, there are two other studies that aimed to classified the conditions of teeth. These studies are described in Section (S1.2.2) of the Supplemental File.

#### 4.2.3. Detection of Prostheses and Restorations

Dental Prostheses are dental appliances that a dentist can use to replace or restore a missing tooth or missing parts of tooth structure, or structures that need to be removed to prevent decay. These various prostheses include fillings, crowns and bridges, all of which may cause pain in the future. There have been four studies conducted to detect different types of crowns and dental materials. The Supplemental File contains an overview of these studies in Section (S1.2.3).

#### 4.2.4. Teeth Numbering and Missing Teeth

An important part of a dentist’s diagnostic process is the evaluation of dental radiographs. The detection and numbering of teeth is part of the interpretation process carried out by a dental expert. Dental implant placement requires the detection of missing teeth regions. There have been nine studies conducted for teeth numbering and detecting missing teeth. There is a brief overview of these studies in Section (S1.2.4) of the Supplemental File.

#### 4.2.5. Detection of Dental Implants

The application of deep learning offers promising performance in computer vision tasks, and is especially suitable for the analysis and recognition of dental images in dental implants [56]. The detection of dental implants has been the subject of eight papers in this systematic review. In the supplemental file, Section (S1.2.5) provides a brief overview of these studies.

#### 4.2.6. Detection of Bone Loss (Osteoporosis) and Bone Age Measurement (BAM)

In clinical practice, peri-implant bone level detection relies on imaging findings. Commonly used imaging modalities include CBCT (2 studies), panoramic radiography (2 studies), and periapical radiography (6 studies). Furthermore, there are four studies available to estimate the age based on different dental images. These studies is summarized in Section (S1.2.6) of the Supplemental File.

#### 4.2.7. Detection of Periodontal Diseases

A periodontal disease is an oral inflammation that affects the gingival tissues as well as the tissues supporting the teeth. Aside from the fact that they cause tooth loss, they are also linked to cardiovascular diseases, diabetes, and rheumatoid arthritis. There are six papers for detection of periodontal diseases included in this review. The Supplemental File contains a summary of this study in Section (S1.2.7).

#### 4.2.8. Detection of Cysts and Tumors

There are six papers for detection of cysts and tumors are included in this review. An overview of this study can be found in Section (S1.2.8) of the Supplemental File.

#### 4.2.9. Supernumerary and Impacted Wisdom Teeth Detection

“Supernumerary teeth” refer to teeth that are not part of the deciduous or permanent teeth series. Five papers are available for the detection of supernumerary and impacted wisdom teeth. Section (S1.2.9) of the Supplemental File provides an overview of these studies.

#### 4.2.10. Detection of Root (Endodontic) Treatment

There are four papers available regarding the detection of root treatment. Endodontic treatment can be adversely affected by an extra root on the distal root of the mandibular (lower jaw) first molar [57]. An overview of these studies is provided in Section (S1.2.10) of the Supplemental File.

#### 4.2.11. Detection of Cephalometric Landmark

A growing role has been played by quantitative cephalometry in clinical diagnosis, treatment, and surgery. It is essential to develop fully automated methods for these procedures in order to ensure that computerized analyses are accurate. In this systematic review, five papers discuss the detection of cephalometric landmarks. The Supplemental File provides a brief overview of these studies in Section (S1.2.11). Table 4 summarizes the main characteristics of the teeth-targeted studies and all outcomes measured in the study.

### 4.3. Different Dental X-Ray Images

Many types of images, especially the X-ray, have been used in the literature [25]. In the dentistry field, there are different types of X-ray detectors: Orthopantomogram (OPG) and Radiovisiography (RVG). The X-ray image produced using the OPG detector shows both the upper and lower teeth in one image. While RVG takes intraoral radiographs which are useful for diagnosing an individual tooth [36]. In general, there are different types of dental X-rays that dentist uses to evaluate the oral health of teeth:

#### 4.3.1. Intraoral X-Rays Images

The most widely used form of dental X-ray in dental clinics. These X-rays give great information about individual teeth, allowing the dentist to track overall dental and jawbone health. In this type of X-ray image, the film is placed inside the mouth of the patient. There are several types of intraoral X-rays, each showing different aspects of teeth: Bitewing X-rays, Periapical X-rays, and Occlusal X-rays.

**Table 4:** Main characteristics of included teeth targeted studies.

Author	Year	Journal Rank (SJR)/ Conference Rank (Qualis)	Variable Measured	Radiography	# of Images	ML/DL Model	Validation Metrics	Values
Velemínská et al. [24]	2013	Q2	Age Estimation	Panoramic X-rays	1393	RBFNN GAME	Accuracy	-
Oktay et al. [31]	2017	Not Yet Assigned	Tooth classification	Panoramic X-rays	105	AlexNet	Accuracy, Precision recall	0.971, 0.987
Miki et al. [19]	2017	Q1	Tooth classification	CBCT	52	AlexNet	Accuracy	0.88
Raith et al. [34]	2017	Q1	Tooth classification	-	-	ANN	Performance	0.93
Jader et al. [32]	2018	B1	Tooth segmentation	Panoramic X-rays	1500	Mask R-CNN	Precision, Accuracy, Recall, F1-score, Specificity	0.98, 0.88, 0.94, 0.84, 0.99
Lee et al. [12]	2018	Q2	Periodontal diseases	Periapical images	1740	VGG-19	Accuracy	81.0%
Moriyama et al. [58]	2019	B4	Periodontal Pockets	Oral images	2625	YOLOv2, MapReduce	Accuracy, True Positive Rate (TPR), False Positive Rate (FPR), AUC	91.7%, 93.2%, 6.8%, 0.917%
Chen et al. [59]	2019	Q1	Teeth numbering/Missing teeth	Periapical images	1250	Faster R-CNN	Recall, Precision	0.728, 0.771
Ariji et al. [28]	2019	Q1	Cysts and Tumors	Panoramic X-rays	210	DetectNet	Intersection over Union (IoU)	0.88
Tuzoff et al. [4]	2019	Q1	Teeth detection /Teeth numbering	Panoramic X-rays	1352	Faster R-CNN and VGG16	Sensitivity, Precision	0.9941, 0.9945
Lee et al. [30]	2019	Q1	Bone Loss	Panoramic X-rays	1500	SC-DCNN, MC-DCNN	AUC	0.9763, 0.9991 and 0.9987, respectively
Vinayaha-lingam et al. [60]	2021	Q1	Teeth classification	Panoramic X-rays	400	MobileNet-V2	Accuracy, Sensitivity, Specifcity, AUC	0.87, 0.86, 0.88, 0.90
Siva-sundaram et al. [61]	2021	Q2	Cysts and Tumors	Panoramic X-rays	–	Modified LeNet	Accuracy, Sensitivity	99.63% 98.3%
Chandr-ashekar et al. [62]	2022	Q1	Teeth segmentation	Panoramic X-rays	1500	Faster R–CNN and YOLOv5	AUC	98.77%
Oztekin et al. [63]	2022	Q2	Prostheses and Restorations	Panoramic X-rays	250	U-Net and YOLOv5	Accuracy	99.81%
Widiasri et al. [64]	2022	Q1	Bone Loss	CBCT	75	3D U-Net	Accuracy	95.3%
Seo et al. [65]	2022	Q2	Age Estimation	Cephalomet-ric projections	900	DeepLabv3 and Inception-ResNet-v2	Accuracy, IoU, F1 scores	0.956, 0.913, 0.895
Atas et al. [66]	2022	ArXiv	Age Estimation	Panoramic X-rays	1332	InceptionV3 and InceptionV3Mixed 04	MAE, RMSE, R2	3.13, 4.77, 87%
Chen et al. [21]	2021	Q1	Teeth classification	3D dental model	–	DCGANs	Accuracy, macro precision, macro-recall, and macro-F1	91.35%, 91.49%, 91.29%, 0.9139
kim et al. [67]	2021	Q1	Cephalo-metric Landmark	CBCT	430	multi-stage CNNs	SDR, MRE	87.10% and 1.03 mm average MRE.
Yu et al. [68]	2022	Q1	Cysts and Tumors	Panoramic X-rays	10,000 healthy images and 872 lesion images	Two-branch network architecture (MoCoV2, U-Net)	Accuracy, Precision, Sensitivity, Specifcity, F1 score	88.72%, 65.81%, 66.56%, 92.66%, 66.14%
Mine et al. [69]	2022	Q1	Supernumerary Teeth	Panoramic X-rays	220	AlexNet, VGG16-TL, InceptionV3-TL	Accuracy, Sensitivity, Specificity, ROC curve	84.0%, 85.0%, 83.0%
Almalki et al. [70]	2022	Q1	Teeth classification	Panoramic X-rays	1200	YOLOv3	Accuracy	99.33%
Xie et al. [71]	2023	Q1	Teeth Segmentation	CBCT	1000	FCOS	Dice index	–
Rubiu et al. [72]	2023	Q2	Teeth Segmentation	Panoramic X-rays	1000	Mask-RCNN	Accuracy, Dice index	98.4%, 0.87
Yilmaz et al. [73]	2023	Q2	Teeth Classification	Panoramic X-rays	–	RCNN	–	–
Yilmaz et al. [73]	2023	Q2	Teeth Classification	Panoramic X-rays	1200	RCNN and YOLO-V4	precision, recall, F1 score	99.90%, 99.18%, 99.54% for YOLO-V4
karaoglu et al. [74]	2023	Q1	Teeth Numbring	Panoramic X-rays	2702	Mask RCNN	precision, recall, F1 score	92.49%, 96.08%, 95.65% and 95.87%
Park et al. [75]	2023	Q1	Dental Implants	Panoramic and Periapical radiographic	156,965	customized DL model	Accuracy, Precision, Recall, F1 score	88.53%, 85.70%, 82.30%, 84.00%
Hong et al. [76]	2023	Q1	Cephalo-metric Landmark	CBCT	500	DQN and DDQN	Accuracy	67.33% and 66.04%
Ayhan et al. [77]	2024	Q1	Teeth detection /Teeth numbering	Bitewing X-ray	1170	Improved YOLOv7	Accuracy, Recall, Specifcity, Precision and F1-Score	0.934, 0.834, 0.961, 0.851, 0.842
Kurtulus et al. [78]	2024	Q2	Dental Implants	Panoramic X-rays	1258	VGG16, ResNet-50, EfficientNet, ConvNeXt	Accuracy, Precision, Recall, F1-score	95.74%, 96.01%, 94.72% 95.22%
Marginean et al. [79]	2024	Q1	Teeth Segmentation	Panoramic X-rays	150	CariSeg	Accuracy, Dice coefficient	99.42%, 68.2%

#### 4.3.2. Extraoral X-Rays Images

Extraoral X-ray images are diagnostic tools used to capture detailed views of the teeth, jaw, and facial structures from outside the mouth, aiding in comprehensive dental assessment and treatment planning. Dentists use various extraoral X-rays, such as Panoramic X-rays, Cephalometric Projections (CP), and Cone-beam Computed Tomography (CBCT). These imaging techniques provide comprehensive views of dental structures, aiding in accurate diagnosis and effective treatment planning. Panoramic X-rays offer a wide view of the jaw and teeth, while CP focuses on the skull and jaw relationships offer insights into the relationships between the jaw and skull, crucial for orthodontic planning. In addition, AI in 3D dental imaging enhances diagnostics and treatment planning by analyzing CBCT scans to accurately identify issues like cavities and fractures. CBCT stands out by providing high-resolution 3D images, allowing for precise diagnosis and treatment planning, particularly in complex cases like implants and orthodontics. These 3D images provide a detailed view of dental structures, aiding in the creation of precise treatment plans for implants and orthodontics by simulating scenarios and predicting outcomes. These advancements in 3D imaging enhance the dentist’s ability to accurately identify and address dental issues, ultimately improving patient outcomes. Automated measurements and AI-generated models improve efficiency and patient communication, while predictive analytics aid in informed decision-making. CBCT provides detailed 3D images, crucial for complex procedures like implants and orthodontics, ensuring precise assessments and interventions.

#### 4.3.3. Oral Photographs

Oral images can be captured with the help of a consumer camera in a cost-effective and simple manner. It has become increasingly common for consumers to carry cameras, including smartphones, which are easy to use and have enhanced functionality [50,80].

#### 4.3.4. Near-Infrared Transillumination

Near-infrared transillumination (TI) is a promising and effective imaging technique for the detection of early teeth lesions (i.e., caries) in real-time without film [41,81]. Increased mineral loss (caries lesion) leads to an increase in scattering and absorption of light. Therefore, caries appears as dark regions because less light reaches the detector [81].

#### 4.3.5. Fluorescent Imaging

Fluorescence occurs when a substance absorbs higher-energy light and then emits light (photons). It is more intense in the dentine than in the enamel in natural teeth, and it has a bluish-white color [82].

#### 4.3.6. 3D Digital Dental Model

In addition to intraoral scanning technology, digital dental models can be obtained through advancements in digital technology. A resinic dental model can then be created using the stereolithographic data collected from the scanner [83]. Table 5 summarizes the different characteristics and usage of X-ray images.

**Table 5:** Main characteristics and usage of dental X-ray images in literature.

Type	Publication Used	Variable Measured	Sample Image	Features
Bitewing X-rays	[37,49,54,77,84,85,86]	Caries detection (posterior initial proximal caries)	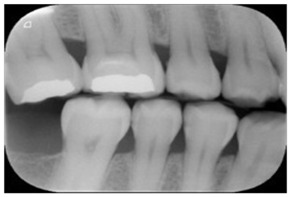	Accuracy
Occlusal X-ray	N.A	Detecting abnormal, extra teeth, jaw fractures, a cleft palate, cysts and abscesses	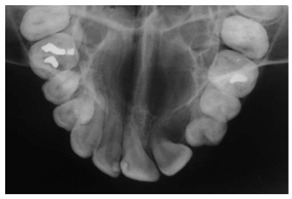	Displaying a section or entire arch of teeth in the upper or lower jaw
Periapical X-rays	[12,39,40,59,86,87,88,89,90,91,92,93]	Diagnosing invisible proximal dental caries	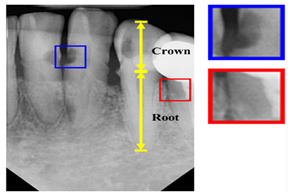	Display the entire tooth, from the crown to the root, where it connects to the jaw.
Radiovisiography (RVG)	[36,42]	Diagnosis of an individual tooth and classification of dental diseases.	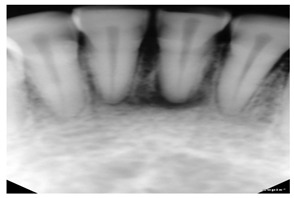	No films placed inside the patient mouth.
Cephalometric projections	[65,94,95,96]	Orthodontic treatment planning. It captures a single film’s anterior, posterior, and lateral image of the skull bones and soft tissues.	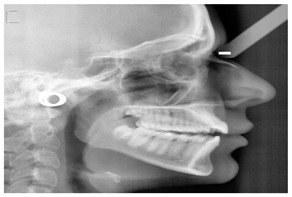	Typically collected from individuals who need orthodontic or orthognathic surgery.
Cone-beam Computed Tomography (CBCT)	[19,38,53,64,67,93,97,98,99,100,101,102,103,104]	Endodontics, orthodontics, implant, oral surgery, and oral medicine	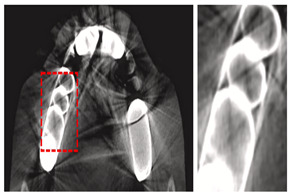	High resolution 3D volumetric data.
Panoramic X-Rays	[4,11,20,22,23,24,26,27,30,31,32,43,44,57,58,60,61,62,63,66,68,69,89,105,106,107,108,109,110,111,112,113,114,115,116,117,118,119,120,121,122,123,124,125,126,127]	Full visualization of jaw, such as tumors, teeth included, infections, post-accident fractures, temporomandibular joint disorders	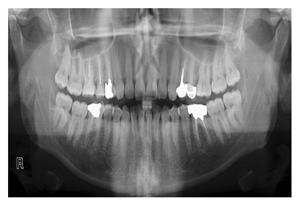	Captured outside the mouth which makes them more acceptable for the patient, they cause a lower infection rate, and lower radiation exposure, they are simple to apply and require less time but they are the most challenging type due to uneven lighting, the presence of noise and low resolution.
Ora Photographs	[50,51,58,89,128,129,130,131]	Gathered by consumer cameras	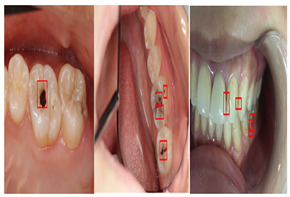	They are easier and more cost-effective to capture.
Near-Infrared Transillumination	[10,41]	Early teeth lesions (i.e., caries) in real time	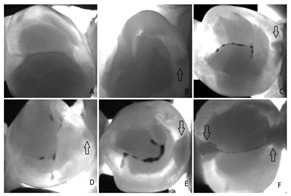	The near-infrared light shows as a dark region in a caries lesion because of light scattering and absorption.
Fluorescent imaging	[132,133]	Identification and analysis of dental plaque to detect disease	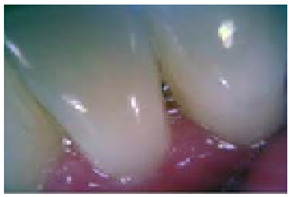	Accuracy
3D digital dental model	[17,18,21,34,106,134]	Planning of treatment in surgery	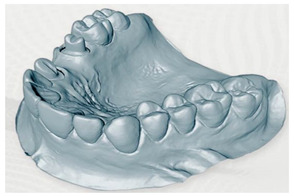	View the dental occlusion in 3D spatial perspective

To conclude, radiographic images are very challenging for the following reasons:

- There are different levels of noise in radiographic images due to the moving imaging device that captures the patient’s teeth.
- The segmentation of objects in panoramic radiographic images can be made difficult by problems such as light imbalances caused by superimposition and other positioning errors [135].

The resolution of panoramic radiographic images is usually low, which contributes to the presence of noise in the image. It is therefore necessary to distinguish between the area of interest (ROI) and the background when processing dental X-ray images [115]. It is important to note that, when compared to other radiographic images, such as intraoral images (bitewing and periapical), these images offer greater patient comfort and provide less radiation exposure to the patient. Additionally, it has ability to examine a larger area of the jaw and maxilla [108].

## 5. Discussion

This study aims to summarize the current state of artificial intelligence’s ability to detect various dental conditions, including dental caries, fillings, endodontic treatment, dental implants, and endodontic treatments. The NN structures vary from single layer to multiple layers with a different number of interconnected nodes, showing different modes of traveling through the network. An increasing interest is being shown in the use of different NN structures, especially for the analysis of medical images. This is because these models are capable of processing large amounts of relevant data for analysis, diagnosis, and surveillance of disease [136].

There has been a general growth in the research that applies AI (specially deep learning) to dentistry fields. Figure 2 shows that the year 2020 followed by year 2021 had the most articles published in this field. This literature review includes studies utilizing a variety of NN architectures, see Figure 3. CNNs are designed to process data that consists of multiple arrays and different backbones. As the detection of dental images has emerged over time, more dense CNNs have been used for this purpose, such as Faster RCNN [137], that utilizes a faster region proposal network (RPN) and a detection network that share convolutional features based on the full-image convolutions. UNet [138] architecture is used to segment images in a fast and precise manner. So far, it has outperformed a sliding-window convolutional network among the most effective methods. Moreover, Compared to the traditional CNN, FCNN [139] improves the computational efficiency and detection accuracy. Some of the convolution layers are weighted directly by Gabor filters [37,40]. The YOLO family [140] architecture is one of the most popular model architectures for detecting objects in real time. The main reason for its popularity is that it utilizes one of the most effective neural network architectures to produce high reliability and efficient processing performance. DetectNet [28] is a deep neural network for detecting objects that provides the XY coordinates of an object detected [27,99,118]. More recent modification of Faster R-CNN is Mask R-CNN [141], which predict segmentation masks for each region of interest (ROI) [126]. Recently, Mask R-CNN and U-net have outperformed other teeth detection and segmentation structures for further teeth diagnosis tasks.

Generally, NNs require large amounts of different types of dental images in order to ensure high levels of targeted accuracy. Overfitting occurs when neural networks learn too well from their training data. So far, NNs cannot be applied to another group of images beyond those trained. This emphasizes the importance of using a variety of data that is matched to a given population. Training on a large amount of data has resulted in very efficient deep CNN algorithms [37,39,50]. Srivastava et al. [37] collect the dataset from approximately 100 clinics across the United States provided them with over 3000 bitewing radiographs, which allowed them to achieve optimal results in finding dental professionals. Lee et al. [39] in their study utilized a total of approximately 3000 periapical radiographs, divided into training and validation sets, where [50] during the development and evaluation of the model, 3932 oral photographs were collected from 625 volunteers with consumer cameras.

In theory, performance of networks with deeper layers is expected to be better than the performance of networks with shallower layers. It appears, however, deep networks perform less well in practice than shallow networks. This is because there was an optimization problem rather than an overfitting problem. To put it simply, the deeper a network is, the more challenging it is to optimize. Therefore, Transfer Learning (TL) is another way to provide a rapid straight-forward progress or improved performance for certain problem such as oral field. Pre-trained Models (AlexNet, GoogLeNet, ResNet, VGG, Inception Networks etc. and more) are an examples of TL that enrich the dentistry diagnostic support system. AlexNet [142] is composed of eight layers, in which five convolutional layers are employed, two hidden layers are fully connected, and a single output layer is fully connected. GoogLeNet [143] has 20 layers and VGG-16 [144] has 16 layers, both trained on ImageNet [142] classifies images into 1000 object categories. Inception [143] is concerned with computation costs, whereas ResNet family [145] is concerned with computation accuracy. As an example of TL, Prajapati et al. [36] and Haghanifar et al. [43] experimented with the performance of CNN for diagnosis by employing transfer learning to classify dental caries.

Alternatively, combining different CNN architectures in one model (hybrid model) shows significant results [38,127]. Using U-net combined with subpixel CNN models resulted in improved quality metrics as well as image segmentation-based analysis compared with techniques for super-resolution reconstruction based on the state-of-the-art [38]. Where [127] utilizes three different U-Net networks with Faster R-CNN and VGG-16 for tooth detection and tooth numbering.

There have been numerous target applications employing NN in the dental field. In our study, we focus on explore the maximum number of teeth target that can be in one research (12 targets). Figure 5a,b demonstrate the emphasis of dental detection in terms of disease or type of radiography, respectively. As can be seen in Figure 5a, teeth caries is the most searched topic [146]. Some approaches focused on the detection of caries in a large [37] or small dataset [36], whereas other suggested a treatment plan based on caries depth [147]. Moreover, teeth segmentation seems to be an effective preprocessing step for further dental disease diagnosis in 2D images [32] or/and 3D teeth models [18,134]. The teeth segmentation aids in distinguishing the teeth from other tissues (i.e., gums and jaw bones). Due to the public availability of datasets, studies have been increasingly focused on measuring the bone level as preprocessing for other treatments (i.e., implant) [30,113] or in measuring the age of bone [24].

There have been variety of data types have been used in the computerized dental targets. In our study, we focus on explore the maximum number of data types that can be used in research (11 types). As can be seen in Figure 5b, Panoramic X-Rays is the most popular data type in literature (with 44.34%) as it provide full visualization of jaw, such as tumors, teeth included, infections, post-accident fractures, temporomandibular joint disorders. This is because it captures outside the mouth which makes them more acceptable for the patient with a lower infection rate, and lower radiation exposure. Also, they are simple to apply and require less time but they are the most challenging type due to uneven lighting, the presence of noise and low resolution (such as [68,69]). CBCT comes in second place (with 12.26%), where it used in endodontics, orthodontics, implant, oral surgery, and oral medicine due to the high resolution 3D volumetric data (such as [64,67]). Then, Periapical X-rays in third place (with 11.32%) for diagnosing invisible proximal dental caries because it displays the entire tooth, from the crown to the root, where it connects to the jaw (such as [40,59]). Recently, the use of Oral Photographs (such as [50,51]) rapidly evolved in recent research from (2019–2022) enabling end-users cameras to capture using mobile applications because it is easier and more cost-effective to capture. A further barrier to setting up training data is the requirement for annotation by medical experts.

Many researcher optimized the performance of their architecture by different techniques such as: augmentation. For example, Miki et al. [19] augmented the data by image rotation and intensity transformation, and Sivasundaram et al. [61] enhanced the number of input samples and performed a threefold cross-validation in order to evaluate the accuracy of the results by using data augmentation and threefold cross-validation. Also, Almalki et al. [70] used it to increase the dataset size, several augmentation functions were used to increase the number of images, including rotation, shear, zooming, and horizontal and vertical flipping. In the other hand, Other diagnoses focus on integrating image analysis tools with dental radiography as pre-processing or post-processing such as [88,148]. Sabharwal et al. [148] reviewed different methods that combine DL with image analysis for implant and periodontal diseases to understand their impact and how this can lead to improved treatment results. Also, Choi et al. in [88] used a preprocessing step (i.e., horizontal alignment of pictured teeth) followed by a fully convolutional network model with Naïve classifier [149]. For post-processsing, Chen et al. [59] proposed three post-processing techniques to improve detection precision of faster R-CNN.

To this end, there are a variety of alternatives available to researchers in dental-care problems. According to our study, we found that there is little guidance in the literature on selecting appropriate methods for each target. Therefore, there is a need to collaborate between dentists and DL developers to clarify the optimal model for each teeth target.

In future clinical applications, hybrid models will be taken into account in order to increase accuracy for each target. It is likely that more TL-based techniques will be applied in the future, especially for more successful techniques (U-net [38,49,101], Mask R-CNN [23,125] and Faster R-CNN [23,125]). Additionally, prediction target networks will probably be seen more in the future, such as [12,147].

## 6. Conclusions

The recognition of dental images has progressively advanced with the introduction of more complex convolutional neural networks (CNNs), achieving significant enhancements in accuracy. As the acquisition of big data grows, the demand for the efficient processing capabilities of deep CNN technologies becomes increasingly critical. Given the substantial diversity in image databases, as well as the variability in types, outcomes, and frameworks of neural networks (NNs), a standardized approach is essential to enhance comparability and robustness across studies. To further advance standardization, generalizability, and reproducibility in dental imaging, future research should focus on identifying the most effective imaging modality for each specific dental application. Additionally, the potential of transfer learning and hybrid models has shown promising results in terms of performance improvement. However, more experimental studies are required to verify their effectiveness across various dental target studies. Future research in dental imaging should focus on developing standardized protocols for image acquisition and processing to enhance comparability across studies. Identifying the most effective imaging modalities for specific dental applications is crucial to improve diagnostic accuracy. Additionally, exploring the potential of transfer learning and hybrid models through experimental studies can ensure their applicability across diverse datasets. Efficient management of big data is essential, emphasizing advanced storage, retrieval, and processing techniques. Robust frameworks that accommodate variability in neural network architectures are needed to ensure consistent performance. Enhancing the generalizability and reproducibility of CNN models should be prioritized, possibly through cross-validation with diverse datasets. Interdisciplinary collaboration between dental researchers, data scientists, and software developers is vital for innovating and refining AI applications in dentistry. AI in dentistry faces challenges such as insufficient data quality and quantity, lack of standardization, and difficulties in model interpretability. Models often struggle with generalizability across diverse datasets and integrating into clinical workflows. There are also ethical and legal concerns, including patient privacy and liability issues. Additionally, high costs and the need for specialized expertise can limit accessibility, while resistance from dental professionals may hinder adoption. Addressing these issues is essential for effective AI integration in dental practices.

### Limitation of Included Research

In systematic review methodology, the use of filters is generally discouraged due to the potential risk of omitting relevant studies. However, in this review, the filters applied did not significantly impact the retrieval of pertinent articles. The limitations were carefully chosen to minimize the inclusion of irrelevant articles without compromising the scope of relevant findings. Specifically, the review was restricted to human studies, and only papers published between 2013 and 2022 were considered. These criteria were deemed appropriate given the focus of the review and are unlikely to have biased the results significantly.

The temporal restriction was particularly considered to reflect recent advancements and current practices, thereby enhancing the review’s relevance to contemporary research and practice in the field. This approach ensured that the most up-to-date and applicable findings were included, providing a modern perspective on the use of neural networks in dental imaging. However, it is acknowledged that this may also limit the historical perspective and exclude seminal works published prior to 2013 that could still be relevant to understanding the full landscape of the field.

## Figures and Tables

**Table 1:** Overview of Databases and Keywords Used in Systematic Review of Diagnostic AI Applications in Dentistry (2013–2024).

Database	Search Strategy	Search Data	# of Identify Records
IEEE Xplore	“Dental OR Oral OR Dental Diseases OR Periodontal Disease OR Tooth Decay & Cavities OR Oral Cancer OR Gums Disease OR Age Estimation OR Bone Loos” AND “Machine learning OR Deep Learning OR Artificial intelligence” OR “Full Text OR Paper Title” OR “Survey” OR “Overview”	2 August 2024	195
Science Direct			608
PubMed (MIDLINE)			3000
arXiv.org			17
MDPI			70
Nature			251
Scopus			1002
Wiley Online Library			85

**Table 2:** Summary of statistical performance indicators used in the analyzed papers. See notes a–f for detailed definitions and additional information.

Metrics	Formula	Definition
Accuracy	$\frac{TP^{a}+TN^{b}}{TP+TN+FP^{c}+FN^{d}}$	The accuracy of a measurement is the degree to which it is close to the true value.
Precision	$\frac{TP}{TP+FP}$	Precision refers to how closely the measurements are related.
Recall (Sensitivity)	$\frac{TP}{TP+FN}$	The recall indicates whether the model is capable of detecting positive samples.
Specificity	$\frac{TN}{TN+FP}$	It is defined as the proportion of true negatives that the model correctly predicts.
F1 score (Dice Coefficient)	$\frac{2\;\cdot \;TP}{2\;\cdot \;TP+FP+FN}$	In the F1 score, the precision and recall are calculated as a harmonic mean.
Jaccard index (Intersection over Union (IoU))	$\frac{TP}{TP+FN+FP}$	A Jaccard similarity coefficient, also known as the Jaccard index, measures the similarity and diversity of sample sets.
Mean Absolute Error (MAE)	$\frac{1}{n}\sum_{i=1}^{n}{\left|(\hat{y_{i}}-y_{i})\right|}^{e}$	It is a measure of the difference in error between pairs of observations expressing the same phenomenon.
Root Mean Square Error (RMSE)	$\frac{1}{n}\sum_{i=1}^{n}{\left(\hat{y_{i}}-y_{i}\right)}^{2}$	Typically refers to the difference between the values predicted by a model or an estimator and the values observed.
Correlation Coefficient (R2)	$\frac{\frac{1}{n}\sum_{i=1}^{n}{\left(\hat{y_{i}}-\underline{y}\right)}^{2}-\frac{1}{n}\sum_{i=1}^{n}{\left(\hat{y_{i}}-y_{i}\right)}^{2}}{\frac{1}{n}\sum_{i=1}^{n}{\left(y_{i}-\underline{y}\right)}^{2}}$	An estimation method based on statistics used to evaluate the performance of a regression model.
Mean Radial Errors (MRE)	${\frac{\sum_{i=1}^{n}R_{i}}{n}}^{f}$	It is the mean Euclidian distance between the reference turning point and the predicted point.
Successful Detection Rate (SDR)	$\frac{\mathrm{number}\;\mathrm{of}\;\mathrm{accurate}\;\mathrm{samples}}{\mathrm{number}\;\mathrm{of}\;\mathrm{samples}}\times 100\%$	When the error between the estimated coordinates and the correct position is less than a precision range, the estimated coordinates are considered correct.

a—TP is true positive. b—TN is true negative. c—FP is false positive. d—FN is false negative. e—The n indicates the total number of samples. *y_i_* refers to the estimated value, while $\hat{y_{i}}$ stands for actual value and $\underline{y}$ demonstrate the true mean value. f—*n* represents the size of the set, where radial error *R* is defined as the distance between the predicted coordinates and the actual coordinates based on the Euclidean distance.

## Data Availability

The data used in this study has been provided in the references of this paper and Supplementary File. PRISMA 2020 Checklist available at: https://shorturl.at/rV8BC (accessed on 22 August 2024).

## Supplementary Materials

The following supporting information can be downloaded at: https://www.mdpi.com/article/10.3390/diagnostics14212442/s1.

## References

[1] Thanh M.T.G., Van Toan N., Ngoc V.T.N., Tra N.T., Giap C.N., Nguyen D.M. (2022). Deep Learning Application in Dental Caries Detection Using Intraoral Photos Taken by Smartphones. Appl. Sci..

[2] Lakshmi M.M., Chitra P. (2020). Classification of Dental Cavities from X-ray images using Deep CNN algorithm. Proceedings of the 2020 4th International Conference on Trends in Electronics and Informatics (ICOEI)(48184).

[3] Ehtesham H., Safdari R., Mansourian A., Tahmasebian S., Mohammadzadeh N., Pourshahidi S. (2019). Developing a new intelligent system for the diagnosis of oral medicine with case-based reasoning approach. Oral Dis..

[4] Tuzoff D.V., Tuzova L.N., Bornstein M.M., Krasnov A.S., Kharchenko M.A., Nikolenko S.I., Sveshnikov M.M., Bednenko G.B. (2019). Tooth detection and numbering in panoramic radiographs using convolutional neural networks. Dentomaxillofac. Radiol..

[5] Topol E.J. (2019). High-performance medicine: The convergence of human and artificial intelligence. Nat. Med..

[6] Mendonça E.A. (2004). Clinical decision support systems: Perspectives in dentistry. J. Dent. Educ..

[7] Tarvonen P.L., Suominen A., Yang G., Ri Y., Sipilä K. (2017). Association between oral health habits and dental caries among children in Pyongyang, Democratic People’s Republic of Korea. Int. J. Dent. Hyg..

[8] Xue Y., Zhang R., Deng Y., Chen K., Jiang T. (2017). A preliminary examination of the diagnostic value of deep learning in hip osteoarthritis. PLoS ONE.

[9] Sklan J.E., Plassard A.J., Fabbri D., Landman B.A. Toward content-based image retrieval with deep convolutional neural networks. Proceedings of the Medical Imaging 2015: Biomedical Applications in Molecular, Structural, and Functional Imaging.

[10] Schwendicke F., Elhennawy K., Paris S., Friebertshäuser P., Krois J. (2020). Deep learning for caries lesion detection in near-infrared light transillumination images: A pilot study. J. Dent..

[11] Krois J., Ekert T., Meinhold L., Golla T., Kharbot B., Wittemeier A., Dörfer C., Schwendicke F. (2019). Deep learning for the radiographic detection of periodontal bone loss. Sci. Rep..

[12] Lee J.H., Kim D.h., Jeong S.N., Choi S.H. (2018). Diagnosis and prediction of periodontally compromised teeth using a deep learning-based convolutional neural network algorithm. J. Periodontal Implant. Sci..

[13] Schwendicke F., Golla T., Dreher M., Krois J. (2019). Convolutional neural networks for dental image diagnostics: A scoping review. J. Dent..

[14] Page M.J., McKenzie J.E., Bossuyt P.M., Boutron I., Hoffmann T.C., Mulrow C.D., Shamseer L., Tetzlaff J.M., Akl E.A., Brennan S.E. (2021). The PRISMA 2020 statement: An updated guideline for reporting systematic reviews. Syst. Rev..

[15] Whiting P.F., Rutjes A.W., Westwood M.E., Mallett S., Deeks J.J., Reitsma J.B., Leeflang M.M., Sterne J.A., Bossuyt P.M., QUADAS-2 Group (2011). QUADAS-2: A revised tool for the quality assessment of diagnostic accuracy studies. Ann. Intern. Med..

[16] Kidd E., Fejerskov O. (2016). Detection, diagnosis, and recording in the clinic. Essentials of Dental Caries.

[17] Sun D., Pei Y., Song G., Guo Y., Ma G., Xu T., Zha H. Tooth segmentation and labeling from digital dental casts. Proceedings of the 2020 IEEE 17th International Symposium on Biomedical Imaging (ISBI).

[18] Tian S., Dai N., Zhang B., Yuan F., Yu Q., Cheng X. (2019). Automatic classification and segmentation of teeth on 3D dental model using hierarchical deep learning networks. IEEE Access.

[19] Miki Y., Muramatsu C., Hayashi T., Zhou X., Hara T., Katsumata A., Fujita H. (2017). Classification of teeth in cone-beam CT using deep convolutional neural network. Comput. Biol. Med..

[20] Muramatsu C., Morishita T., Takahashi R., Hayashi T., Nishiyama W., Ariji Y., Zhou X., Hara T., Katsumata A., Ariji E. (2021). Tooth detection and classification on panoramic radiographs for automatic dental chart filing: Improved classification by multi-sized input data. Oral Radiol..

[21] Chen Q., Huang J., Salehi H.S., Zhu H., Lian L., Lai X., Wei K. (2021). Hierarchical CNN-based occlusal surface morphology analysis for classifying posterior tooth type using augmented images from 3D dental surface models. Comput. Methods Programs Biomed..

[22] Muresan M.P., Barbura A.R., Nedevschi S. Teeth Detection and Dental Problem Classification in Panoramic X-Ray Images using Deep Learning and Image Processing Techniques. Proceedings of the 2020 IEEE 16th International Conference on Intelligent Computer Communication and Processing (ICCP).

[23] Başaran M., Çelik Ö., Bayrakdar I.S., Bilgir E., Orhan K., Odabaş A., Aslan A.F., Jagtap R. (2021). Diagnostic charting of panoramic radiography using deep-learning artificial intelligence system. Oral Radiol..

[24] Velemínská J., Pílnỳ A., Cepek M., Kot’ová M., Kubelková R. (2013). Dental age estimation and different predictive ability of various tooth types in the Czech population: Data mining methods. Anthropol. Anzeiger; Ber. Uber Die Biol.-Anthropol. Lit..

[25] Kumar A., Bhadauria H.S., Singh A. (2021). Descriptive analysis of dental X-ray images using various practical methods: A review. PeerJ Comput. Sci..

[26] Ekert T., Krois J., Meinhold L., Elhennawy K., Emara R., Golla T., Schwendicke F. (2019). Deep learning for the radiographic detection of apical lesions. J. Endod..

[27] Fukuda M., Inamoto K., Shibata N., Ariji Y., Yanashita Y., Kutsuna S., Nakata K., Katsumata A., Fujita H., Ariji E. (2020). Evaluation of an artificial intelligence system for detecting vertical root fracture on panoramic radiography. Oral Radiol..

[28] Ariji Y., Yanashita Y., Kutsuna S., Muramatsu C., Fukuda M., Kise Y., Nozawa M., Kuwada C., Fujita H., Katsumata A. (2019). Automatic detection and classification of radiolucent lesions in the mandible on panoramic radiographs using a deep learning object detection technique. Oral Surg. Oral Med. Oral Pathol. Oral Radiol..

[29] Lee J.H., Kim D.H., Jeong S.N. (2020). Diagnosis of cystic lesions using panoramic and cone beam computed tomographic images based on deep learning neural network. Oral Dis..

[30] Lee J.S., Adhikari S., Liu L., Jeong H.G., Kim H., Yoon S.J. (2019). Osteoporosis detection in panoramic radiographs using a deep convolutional neural network-based computer-assisted diagnosis system: A preliminary study. Dentomaxillofacial Radiol..

[31] Oktay A.B. Tooth detection with convolutional neural networks. Proceedings of the 2017 Medical Technologies National Congress (TIPTEKNO).

[32] Jader G., Fontineli J., Ruiz M., Abdalla K., Pithon M., Oliveira L. Deep instance segmentation of teeth in panoramic X-ray images. Proceedings of the Conference on Graphics, Patterns and Images (SIBGRAPI).

[33] Ali R.B., Ejbali R., Zaied M. Detection and classification of dental caries in X-ray images using deep neural networks. Proceedings of the ICSEA 2016: The Eleventh International Conference on Software Engineering Advances.

[34] Raith S., Vogel E.P., Anees N., Keul C., Güth J.F., Edelhoff D., Fischer H. (2017). Artificial Neural Networks as a powerful numerical tool to classify specific features of a tooth based on 3D scan data. Comput. Biol. Med..

[35] Forouzeshfar P., Safaei A.A., Ghaderi F., Hashemi Kamangar S., Kaviani H., Haghi S. (2024). Dental caries diagnosis using neural networks and deep learning: A systematic review. Multimed. Tools Appl..

[36] Prajapati S.A., Nagaraj R., Mitra S. Classification of dental diseases using CNN and transfer learning. Proceedings of the International Symposium on Computational and Business Intelligence (ISCBI).

[37] Srivastava M.M., Kumar P., Pradhan L., Varadarajan S. (2017). Detection of tooth caries in bitewing radiographs using deep learning. arXiv.

[38] Hatvani J., Horváth A., Michetti J., Basarab A., Kouamé D., Gyöngy M. (2018). Deep learning-based super-resolution applied to dental computed tomography. IEEE Trans. Radiat. Plasma Med. Sci..

[39] Lee J.H., Kim D.H., Jeong S.N., Choi S.H. (2018). Detection and diagnosis of dental caries using a deep learning-based convolutional neural network algorithm. J. Dent..

[40] Zhang K., Wu J., Chen H., Lyu P. (2018). An effective teeth recognition method using label tree with cascade network structure. Comput. Med. Imaging Graph..

[41] Casalegno F., Newton T., Daher R., Abdelaziz M., Lodi-Rizzini A., Schürmann F., Krejci I., Markram H. (2019). Caries detection with near-infrared transillumination using deep learning. J. Dent. Res..

[42] Geetha V., Aprameya K., Hinduja D.M. (2020). Dental caries diagnosis in digital radiographs using back-propagation neural network. Health Inf. Sci. Syst..

[43] Haghanifar A., Majdabadi M.M., Ko S.B. (2020). Paxnet: Dental caries detection in panoramic X-ray using ensemble transfer learning and capsule classifier. arXiv.

[44] Lee J.H., Han S.S., Kim Y.H., Lee C., Kim I. (2020). Application of a fully deep convolutional neural network to the automation of tooth segmentation on panoramic radiographs. Oral Surg. Oral Med. Oral Pathol. Oral Radiol..

[45] Sonavane A., Yadav R., Khamparia A. (2021). Dental cavity classification of using convolutional neural network. IOP Conf. Ser. Mater. Sci. Eng..

[46] Bui T.H., Hamamoto K., Paing M.P. (2021). Deep fusion feature extraction for caries detection on dental panoramic radiographs. Appl. Sci..

[47] Ding B., Zhang Z., Liang Y., Wang W., Hao S., Meng Z., Guan L., Hu Y., Guo B., Zhao R. (2021). Detection of dental caries in oral photographs taken by mobile phones based on the YOLOv3 algorithm. Ann. Transl. Med..

[48] Zheng L., Wang H., Mei L., Chen Q., Zhang Y., Zhang H. (2021). Artificial intelligence in digital cariology: A new tool for the diagnosis of deep caries and pulpitis using convolutional neural networks. Ann. Transl. Med..

[49] Cantu A.G., Gehrung S., Krois J., Chaurasia A., Rossi J.G., Gaudin R., Elhennawy K., Schwendicke F. (2020). Detecting caries lesions of different radiographic extension on bitewings using deep learning. J. Dent..

[50] Zhang X., Liang Y., Li W., Liu C., Gu D., Sun W., Miao L. (2022). Development and evaluation of deep learning for screening dental caries from oral photographs. Oral Dis..

[51] Kühnisch J., Meyer O., Hesenius M., Hickel R., Gruhn V. (2022). Caries detection on intraoral images using artificial intelligence. J. Dent. Res..

[52] Dayı B., Üzen H., Çiçek İ.B., Duman Ş.B. (2023). A Novel Deep Learning-Based Approach for Segmentation of Different Type Caries Lesions on Panoramic Radiographs. Diagnostics.

[53] Esmaeilyfard R., Bonyadifard H., Paknahad M. (2024). Dental Caries Detection and Classification in CBCT Images Using Deep Learning. Int. Dent. J..

[54] Chaves E.T., Vinayahalingam S., van Nistelrooij N., Xi T., Romero V.H.D., Flügge T., Saker H., Kim A., da Silveira Lima G., Loomans B. (2024). Detection of caries around restorations on bitewings using deep learning. J. Dent..

[55] Chen X., Ma N., Xu T., Xu C. (2024). Deep learning-based tooth segmentation methods in medical imaging: A review. PRoceedings Inst. Mech. Eng. Part H J. Eng. Med..

[56] Chaurasia A., Namachivayam A., Koca-Ünsal R.B., Lee J.H. (2024). Deep-learning performance in identifying and classifying dental implant systems from dental imaging: A systematic review and meta-analysis. J. Periodontal Implant. Sci..

[57] Hiraiwa T., Ariji Y., Fukuda M., Kise Y., Nakata K., Katsumata A., Fujita H., Ariji E. (2019). A deep-learning artificial intelligence system for assessment of root morphology of the mandibular first molar on panoramic radiography. Dentomaxillofac. Radiol..

[58] Moriyama Y., Lee C., Date S., Kashiwagi Y., Narukawa Y., Nozaki K., Murakami S. A MapReduce-like Deep Learning Model for the Depth Estimation of Periodontal Pockets. Proceedings of the HEALTHINF.

[59] Chen H., Zhang K., Lyu P., Li H., Zhang L., Wu J., Lee C.H. (2019). A deep learning approach to automatic teeth detection and numbering based on object detection in dental periapical films. Sci. Rep..

[60] Vinayahalingam S., Kempers S., Limon L., Deibel D., Maal T., Hanisch M., Bergé S., Xi T. (2021). Classification of caries in third molars on panoramic radiographs using deep learning. Sci. Rep..

[61] Sivasundaram S., Pandian C. (2021). Performance analysis of classification and segmentation of cysts in panoramic dental images using convolutional neural network architecture. Int. J. Imaging Syst. Technol..

[62] Chandrashekar G., AlQarni S., Bumann E.E., Lee Y. (2022). Collaborative deep learning model for tooth segmentation and identification using panoramic radiographs. Comput. Biol. Med..

[63] Oztekin F., Katar O., Sadak F., Aydogan M., Yildirim T.T., Plawiak P., Yildirim O., Talo M., Karabatak M. (2022). Automatic semantic segmentation for dental restorations in panoramic radiography images using U-Net model. Int. J. Imaging Syst. Technol..

[64] Widiasri M., Arifin A.Z., Suciati N., Fatichah C., Astuti E.R., Indraswari R., Putra R.H., Za’in C. (2022). Dental-YOL: Alveolar Bone and Mandibular Canal Detection on Cone Beam Computed Tomography Images for Dental Implant Planning. IEEE Access.

[65] Seo H., Hwang J., Jung Y.H., Lee E., Nam O.H., Shin J. (2023). Deep focus approach for accurate bone age estimation from lateral cephalogram. J. Dent. Sci..

[66] Atas I., Ozdemir C., Atas M., Dogan Y. (2022). Forensic Dental Age Estimation Using Modified Deep Learning Neural Network. arXiv.

[67] Kim M.J., Liu Y., Oh S.H., Ahn H.W., Kim S.H., Nelson G. (2021). Automatic cephalometric landmark identification system based on the multi-stage convolutional neural networks with CBCT combination images. Sensors.

[68] Yu D., Hu J., Feng Z., Song M., Zhu H. (2022). Deep learning based diagnosis for cysts and tumors of jaw with massive healthy samples. Sci. Rep..

[69] Mine Y., Iwamoto Y., Okazaki S., Nakamura K., Takeda S., Peng T.Y., Mitsuhata C., Kakimoto N., Kozai K., Murayama T. (2022). Detecting the presence of supernumerary teeth during the early mixed dentition stage using deep learning algorithms: A pilot study. Int. J. Paediatr. Dent..

[70] Almalki Y.E., Din A.I., Ramzan M., Irfan M., Aamir K.M., Almalki A., Alotaibi S., Alaglan G., Alshamrani H.A., Rahman S. (2022). Deep Learning Models for Classification of Dental Diseases Using Orthopantomography X-ray OPG Images. Sensors.

[71] Xie R., Yang Y., Chen Z. (2023). WITS: Weakly-supervised individual tooth segmentation model trained on box-level labels. Pattern Recognit..

[72] Rubiu G., Bologna M., Cellina M., Cè M., Sala D., Pagani R., Mattavelli E., Fazzini D., Ibba S., Papa S. (2023). Teeth Segmentation in Panoramic Dental X-ray Using Mask Regional Convolutional Neural Network. Appl. Sci..

[73] Yilmaz S., Tasyurek M., Amuk M., Celik M., Canger E.M. (2024). Developing deep learning methods for classification of teeth in dental panoramic radiography. Oral Surg. Oral Med. Oral Pathol. Oral Radiol..

[74] Karaoglu A., Ozcan C., Pekince A., Yasa Y. (2023). Numbering teeth in panoramic images: A novel method based on deep learning and heuristic algorithm. Eng. Sci. Technol. Int. J..

[75] Park W., Huh J.K., Lee J.H. (2023). Automated deep learning for classification of dental implant radiographs using a large multi-center dataset. Sci. Rep..

[76] Hong W., Kim S.M., Choi J., Ahn J., Paeng J.Y., Kim H. (2023). Automated Cephalometric Landmark Detection Using Deep Reinforcement Learning. J. Craniofacial Surg..

[77] Ayhan B., Ayan E., Bayraktar Y. (2024). A novel deep learning-based perspective for tooth numbering and caries detection. Clin. Oral Investig..

[78] Kurtulus I.L., Lubbad M., Yilmaz O.M.D., Kilic K., Karaboga D., Basturk A., Akay B., Nalbantoglu U., Yilmaz S., Ayata M. (2024). A robust deep learning model for the classification of dental implant brands. J. Stomatol. Oral Maxillofac. Surg..

[79] Marginean A.C., Mureşan S., Hedeşiu M., Dioşan L. (2024). Teeth Segmentation and Carious Lesions Segmentation in Panoramic X-Ray Images using CariSeg, a Networks’ Ensemble. Heliyon.

[80] Lee J.H. (2016). Future of the smartphone for patients and healthcare providers. Healthc. Inform. Res..

[81] Karlsson L., Maia A.M.A., Kyotoku B.B., Tranaeus S., Gomes A.S.L., Margulis W. (2010). Near-infrared transillumination of teeth: Measurement of a system performance. J. Biomed. Opt..

[82] Volpato C.A.M., Pereira M.R.C., Silva F.S. (2018). Fluorescence of natural teeth and restorative materials, methods for analysis and quantification: A literature review. J. Esthet. Restor. Dent..

[83] Morris R.S., Hoye L.N., Elnagar M.H., Atsawasuwan P., Galang-Boquiren M.T., Caplin J., Viana G.C., Obrez A., Kusnoto B. (2019). Accuracy of Dental Monitoring 3D digital dental models using photograph and video mode. Am. J. Orthod. Dentofac. Orthop..

[84] Lee S., Oh S.i., Jo J., Kang S., Shin Y., Park J.w. (2021). Deep Learning for Early Dental Caries Detection in Bitewing Radiographs. Sci. Rep..

[85] Moran M., Faria M., Giraldi G., Bastos L., Oliveira L., Conci A. (2021). Classification of Approximal Caries in Bitewing Radiographs Using Convolutional Neural Networks. Sensors.

[86] Karatas O., Cakir N.N., Ozsariyildiz S.S., Kis H.C., Demirbuga S., Gurgan C.A. (2021). A deep learning approach to dental restoration classification from bitewing and periapical radiographs. Quintessence Int..

[87] Huang P.W., Huang P.Y., Lin P.L., Hsu H.C. Alveolar bone-loss area detection in periodontitis radiographs using hybrid of intensity and texture analyzed based on FBM model. Proceedings of the 2014 International Conference on Machine Learning and Cybernetics.

[88] Choi J., Eun H., Kim C. (2018). Boosting proximal dental caries detection via combination of variational methods and convolutional neural network. J. Signal Process. Syst..

[89] Lee J.H., Kim Y.T., Lee J.B., Jeong S.N. (2020). A performance comparison between automated deep learning and dental professionals in classification of dental implant systems from dental imaging: A multi-center study. Diagnostics.

[90] Cha J.Y., Yoon H.I., Yeo I.S., Huh K.H., Han J.S. (2021). Peri-implant bone loss measurement using a region-based convolutional neural network on dental periapical radiographs. J. Clin. Med..

[91] Moran M., Faria M., Giraldi G., Bastos L., Conci A. (2021). Do Radiographic Assessments of Periodontal Bone Loss Improve with Deep Learning Methods for Enhanced Image Resolution?. Sensors.

[92] Liu M., Wang S., Chen H., Liu Y. (2022). A pilot study of a deep learning approach to detect marginal bone loss around implants. BMC Oral Health.

[93] Mori M., Ariji Y., Fukuda M., Kitano T., Funakoshi T., Nishiyama W., Kohinata K., Iida Y., Ariji E., Katsumata A. (2022). Performance of deep learning technology for evaluation of positioning quality in periapical radiography of the maxillary canine. Oral Radiol..

[94] Arik S.Ö., Ibragimov B., Xing L. (2017). Fully automated quantitative cephalometry using convolutional neural networks. J. Med. Imaging.

[95] Qian J., Cheng M., Tao Y., Lin J., Lin H. CephaNet: An improved faster R-CNN for cephalometric landmark detection. Proceedings of the 2019 IEEE 16th International Symposium on Biomedical Imaging (ISBI 2019).

[96] Song Y., Qiao X., Iwamoto Y., Chen Y.w. (2020). Automatic cephalometric landmark detection on X-ray images using a deep-learning method. Appl. Sci..

[97] Okada K., Rysavy S., Flores A., Linguraru M.G. (2015). Noninvasive differential diagnosis of dental periapical lesions in cone-beam CT scans. Med. Phys..

[98] Dutra K.L., Haas L., Porporatti A.L., Flores-Mir C., Santos J.N., Mezzomo L.A., Correa M., Canto G.D.L. (2016). Diagnostic accuracy of cone-beam computed tomography and conventional radiography on apical periodontitis: A systematic review and meta-analysis. J. Endod..

[99] Ariji Y., Fukuda M., Kise Y., Nozawa M., Yanashita Y., Fujita H., Katsumata A., Ariji E. (2019). Contrast-enhanced computed tomography image assessment of cervical lymph node metastasis in patients with oral cancer by using a deep learning system of artificial intelligence. Oral Surg. Oral Med. Oral Pathol. Oral Radiol..

[100] Chung M., Lee M., Hong J., Park S., Lee J., Lee J., Yang I.H., Lee J., Shin Y.G. (2020). Pose-aware instance segmentation framework from cone beam CT images for tooth segmentation. Comput. Biol. Med..

[101] Zheng Z., Yan H., Setzer F.C., Shi K.J., Mupparapu M., Li J. (2020). Anatomically constrained deep learning for automating dental cbct segmentation and lesion detection. IEEE Trans. Autom. Sci. Eng..

[102] Yang Y., Xie R., Jia W., Chen Z., Yang Y., Xie L., Jiang B. (2021). Accurate and automatic tooth image segmentation model with deep convolutional neural networks and level set method. Neurocomputing.

[103] Kurt Bayrakdar S., Orhan K., Bayrakdar I.S., Bilgir E., Ezhov M., Gusarev M., Shumilov E. (2021). A deep learning approach for dental implant planning in cone-beam computed tomography images. BMC Med. Imaging.

[104] Orhan K., Bilgir E., Bayrakdar I.S., Ezhov M., Gusarev M., Shumilov E. (2021). Evaluation of artificial intelligence for detecting impacted third molars on cone-beam computed tomography scans. J. Stomatol. Oral Maxillofac. Surg..

[105] ALbahbah A.A., El-Bakry H.M., Abd-Elgahany S. (2016). Detection of caries in panoramic dental X-ray images using back-propagation neural network. Int. J. Electron. Commun. Comput. Eng..

[106] Naam J., Harlan J., Madenda S., Wibowo E.P. (2016). The algorithm of image edge detection on panoramic dental X-ray using multiple morphological gradient (mmg) method. Int. J. Adv. Sci. Eng. Inf. Technol..

[107] Avuçlu E., Başçiftçi F. (2019). Novel approaches to determine age and gender from dental X-ray images by using multiplayer perceptron neural networks and image processing techniques. Chaos Solitons Fractals.

[108] Kim J., Lee H.S., Song I.S., Jung K.H. (2019). DeNTNet: Deep Neural Transfer Network for the detection of periodontal bone loss using panoramic dental radiographs. Sci. Rep..

[109] Vinayahalingam S., Xi T., Bergé S., Maal T., de Jong G. (2019). Automated detection of third molars and mandibular nerve by deep learning. Sci. Rep..

[110] Zhao Y., Li P., Gao C., Liu Y., Chen Q., Yang F., Meng D. (2020). TSASNet: Tooth segmentation on dental panoramic X-ray images by Two-Stage Attention Segmentation Network. Knowl.-Based Syst..

[111] Kim C., Kim D., Jeong H., Yoon S.J., Youm S. (2020). Automatic tooth detection and numbering using a combination of a CNN and heuristic algorithm. Appl. Sci..

[112] Sukegawa S., Yoshii K., Hara T., Yamashita K., Nakano K., Yamamoto N., Nagatsuka H., Furuki Y. (2020). Deep neural networks for dental implant system classification. Biomolecules.

[113] Chang H.J., Lee S.J., Yong T.H., Shin N.Y., Jang B.G., Kim J.E., Huh K.H., Lee S.S., Heo M.S., Choi S.C. (2020). Deep learning hybrid method to automatically diagnose periodontal bone loss and stage periodontitis. Sci. Rep..

[114] Sharifonnasabi F., Jhanjhi N., John J., Alaboudi A., Nambiar P. (2020). A Review on Automated Bone Age Measurement Based on Dental OPG Images. Int. J. Eng. Res. Technol..

[115] Kahaki S.M., Nordin M., Ahmad N.S., Arzoky M., Ismail W. (2020). Deep convolutional neural network designed for age assessment based on orthopantomography data. Neural Comput. Appl..

[116] Thanathornwong B., Suebnukarn S. (2020). Automatic detection of periodontal compromised teeth in digital panoramic radiographs using faster regional convolutional neural networks. Imaging Sci. Dent..

[117] Kwon O., Yong T.H., Kang S.R., Kim J.E., Huh K.H., Heo M.S., Lee S.S., Choi S.C., Yi W.J. (2020). Automatic diagnosis for cysts and tumors of both jaws on panoramic radiographs using a deep convolution neural network. Dentomaxillofac. Radiol..

[118] Kuwada C., Ariji Y., Fukuda M., Kise Y., Fujita H., Katsumata A., Ariji E. (2020). Deep learning systems for detecting and classifying the presence of impacted supernumerary teeth in the maxillary incisor region on panoramic radiographs. Oral Surg. Oral Med. Oral Pathol. Oral Radiol..

[119] Lian L., Zhu T., Zhu F., Zhu H. (2021). Deep Learning for Caries Detection and Classification. Diagnostics.

[120] Cui W., Zeng L., Chong B., Zhang Q. Toothpix: Pixel-Level Tooth Segmentation in Panoramic X-Ray Images based on Generative Adversarial Networks. Proceedings of the 2021 IEEE 18th International Symposium on Biomedical Imaging (ISBI).

[121] Leite A.F., Gerven A.V., Willems H., Beznik T., Lahoud P., Gaêta-Araujo H., Vranckx M., Jacobs R. (2021). Artificial intelligence-driven novel tool for tooth detection and segmentation on panoramic radiographs. Clin. Oral Investig..

[122] Kılıc M.C., Bayrakdar I.S., Çelik Ö., Bilgir E., Orhan K., Aydın O.B., Kaplan F.A., Sağlam H., Odabaş A., Aslan A.F. (2021). Artificial intelligence system for automatic deciduous tooth detection and numbering in panoramic radiographs. Dentomaxillofac. Radiol..

[123] Lin S.Y., Chang H.Y. (2021). Tooth Numbering and Condition Recognition on Dental Panoramic Radiograph Images Using CNNs. IEEE Access.

[124] Sukegawa S., Yoshii K., Hara T., Matsuyama T., Yamashita K., Nakano K., Takabatake K., Kawai H., Nagatsuka H., Furuki Y. (2021). Multi-task deep learning model for classification of dental implant brand and treatment stage using dental panoramic radiograph images. Biomolecules.

[125] Imak A., Çelebi A., Türkoğlu M., Şengür A. (2022). Dental Material Detection based on Faster Regional Convolutional Neural Networks and Shape Features. Neural Process. Lett..

[126] Park J., Lee J., Moon S., Lee K. (2022). Deep Learning Based Detection of Missing Tooth Regions for Dental Implant Planning in Panoramic Radiographic Images. Applied Sciences.

[127] Estai M., Tennant M., Gebauer D., Brostek A., Vignarajan J., Mehdizadeh M., Saha S. (2022). Deep learning for automated detection and numbering of permanent teeth on panoramic images. Dentomaxillofacial Radiol..

[128] Lerner H., Mouhyi J., Admakin O., Mangano F. (2020). Artificial intelligence in fixed implant prosthodontics: A retrospective study of 106 implant-supported monolithic zirconia crowns inserted in the posterior jaws of 90 patients. BMC Oral Health.

[129] Alalharith D.M., Alharthi H.M., Alghamdi W.M., Alsenbel Y.M., Aslam N., Khan I.U., Shahin S.Y., Dianišková S., Alhareky M.S., Barouch K.K. (2020). A deep learning-based approach for the detection of early signs of gingivitis in orthodontic patients using faster region-based convolutional neural networks. Int. J. Environ. Res. Public Health.

[130] Takahashi T., Nozaki K., Gonda T., Mameno T., Ikebe K. (2021). Deep learning-based detection of dental prostheses and restorations. Sci. Rep..

[131] Warin K., Limprasert W., Suebnukarn S., Jinaporntham S., Jantana P. (2021). Automatic classification and detection of oral cancer in photographic images using deep learning algorithms. J. Oral Pathol. Med..

[132] Imangaliyev S., Veen M.H., Volgenant C., Keijser B.J., Crielaard W., Levin E. Deep learning for classification of dental plaque images. Proceedings of the International Workshop on Machine Learning, Optimization, and Big Data.

[133] Yauney G., Angelino K., Edlund D., Shah P. Convolutional neural network for combined classification of fluorescent biomarkers and expert annotations using white light images. Proceedings of the 2017 IEEE 17th International Conference on Bioinformatics and Bioengineering (BIBE).

[134] Xu X., Liu C., Zheng Y. (2018). 3D tooth segmentation and labeling using deep convolutional neural networks. IEEE Trans. Vis. Comput. Graph..

[135] Akarslan Z.Z., Erten H., Güngör K., Celik I. (2003). Common errors on panoramic radiographs taken in a dental school. J. Contemp. Dent. Pract..

[136] Prados-Privado M., Villalón J.G., Martínez-Martínez C.H., Ivorra C. (2020). Dental images recognition technology and applications: A literature review. Appl. Sci..

[137] Ren S., He K., Girshick R., Sun J. (2015). Faster r-cnn: Towards real-time object detection with region proposal networks. IEEE Trans. Pattern Anal. Mach. Intell..

[138] Ronneberger O., Fischer P., Brox T. (2015). U-net: Convolutional networks for biomedical image segmentation. Proceedings of the International Conference on Medical Image Computing and Computer-Assisted Intervention.

[139] Long J., Shelhamer E., Darrell T. Fully convolutional networks for semantic segmentation. Proceedings of the IEEE Conference on Computer Vision and Pattern Recognition.

[140] Redmon J., Divvala S., Girshick R., Farhadi A. You only look once: Unified, real-time object detection. Proceedings of the IEEE Conference on Computer Vision and Pattern Recognition.

[141] He K., Gkioxari G., Dollár P., Girshick R. Mask r-cnn. Proceedings of the IEEE International Conference on Computer Vision.

[142] Krizhevsky A., Sutskever I., Hinton G.E. (2017). Imagenet classification with deep convolutional neural networks. Commun. ACM.

[143] Szegedy C., Liu W., Jia Y., Sermanet P., Reed S., Anguelov D., Erhan D., Vanhoucke V., Rabinovich A. Going deeper with convolutions. Proceedings of the IEEE Conference on Computer Vision and Pattern Recognition.

[144] Simonyan K., Zisserman A. (2014). Very deep convolutional networks for large-scale image recognition. arXiv.

[145] He K., Zhang X., Ren S., Sun J. Deep residual learning for image recognition. Proceedings of the IEEE Conference on Computer Vision and Pattern Recognition.

[146] Selwitz R.H., Ismail A.I., Pitts N.B. (2007). Dental caries. Lancet.

[147] Bouchahma M., Hammouda S.B., Kouki S., Alshemaili M., Samara K. An automatic dental decay treatment prediction using a deep convolutional neural network on X-ray images. Proceedings of the 2019 IEEE/ACS 16th International Conference on Computer Systems and Applications (AICCSA).

[148] Sabharwal A., Kavthekar N., Miecznikowski J., Glogauer M., Maddi A., Sarder P. (2022). Integrating Image Analysis and Dental Radiography for Periodontal and Peri-Implant Diagnosis. Front. Dent. Med..

[149] Rish I. An empirical study of the naive Bayes classifier. Proceedings of the IJCAI 2001 Workshop on Empirical Methods in Artificial Intelligence.

