# Supplementary material for: Exploring the Applications of Artificial Intelligence in Dental Image Detection: A Systematic Review
Source: Diagnostics (Basel). 2024 Oct 31;14(21):2442. doi: 10.3390/diagnostics14212442 (PMC11545562; doi:10.3390/diagnostics14212442)
Supplement: Supplementary file 1 [file diagnostics-14-02442-s001.zip › diagnostics-3195983-supplementary.pdf]

diagnostics

Supplementary Materials

Exploring the Applications of Artificial Intelligence in Dental Image Detection: A Systematic Review

Shuaa S. Alharbi * and Haifa F. Alhasson

Department of Information Technology, College of Computer, Qassim University, Buraydah 52571, Saudi Arabia; hhson@qu.edu.sa
* Correspondence: shuaa.s.alharbi@qu.edu.sa

1. Machine Learning/ Deep Learning for Dental Disease Detection

Currently, there is a growing interest in applying Artificial Intelligent (AI) strategies and image processing for medical image classification, detection, segmentation, and analysis. Generally, many dental applications and different modalities are used in dental imaging [1]. Some researchers design applications for specific types of dental diseases, while others focus on distinguishing and recognizing different variables, such as distinguishing the teeth from other tissues.

1.1. Caries Targeted Studies

Early detection of dental caries (a.k.a cavity) can prevent tooth damage and save expensive healthcare costs. Thus, an effective modality for the early detection of dental caries is a crucial subject in dental research [2]. From 2015 to 2024, twenty four studies were conducted on dental caries.

The early method by Ali *et al.* in [3] aimed to analyze dental x-ray images and classify them into normal or decayed teeth. They proposed a system for the detection and classification of dental caries in X-ray images using a deep NN by utilizing two different classifier: softmax and stacked sparse auto-encoder [4].

Other methods improved the detection of the cavities based on training using a large amount of data. Using a large amount of data, Srivastava *et al.* [5] developed a fully deep CNN [6] that utilizes 100+ layers to detect caries on bitewing radiographs. Cantu *et al.* [7] aimed to apply deep U-net to detect caries lesions of different radiographic extension on bitewings. In work of Lee *et al.* [8], a deep CNN algorithms were evaluated for AI effectiveness in detecting dental caries on periapical radiographs. Sonavane *et al.* [9] exploblack using a Convolutional Neural Network (CNN) for classifying dental cavities. It demonstrated how CNNs improved accuracy and efficiency in identifying different types of cavities, highlighting potential advancements in dental diagnostics through automated image analysis. This study utilized visual images of teeth and applied a deep convolutional neural network (CNN) to classify them as caries or non-caries. Using images from a Kaggle dataset, the model achieved an accuracy of 71.43%. Bui *et al.* [10] described a method that improved caries detection on dental panoramic radiographs using deep fusion feature extraction. By integrating multiple deep learning techniques, the approach enhanced accuracy in identifying dental caries, providing a more reliable diagnostic tool for dental professionals. The paper addressed the widespread issue of caries affecting billions globally and noted the limitations in existing detection methods. It proposed a computer-aided diagnosis method to detect caries using dental radiographs. The approach involved two main processes: feature extraction and classification. In the feature extraction phase, a 2D tooth image was used to extract deep activated and geometric features. These were combined into a fusion feature set. This set was then tested with various classification models, including SVM, KNN, decision tree, Naïve Bayes, and random forest, to find the best fit. Ding *et al.* [11] used oral photographs taken with mobile phones from 570 patients to create

three datasets: augmented images (n=3,990), enhanced images (n=3,990), and a combination of both (n=7,980). An independent test set was composed of images from another 70 patients. The YOLOv3 network was employed for transfer learning to model the data. Diagnostic precision, recall, F1-score, and mean average precision (mAP) were calculated to evaluate the detection and diagnostic performance of the YOLOv3 algorithm. Zheng *et al.* [12] proposed study that included 844 radiographs, with 717 (85%) used for training and 127 (15%) for testing three convolutional neural networks (CNNs): VGG19, Inception V3, and ResNet18. The performance metrics—accuracy, precision, sensitivity, specificity, and area under the receiver operating characteristic curve (AUC)—were evaluated and compablack. The best-performing CNN model was integrated with clinical parameters to assess if a multi-modal CNN could enhance performance. The Gradient-weighted Class Activation Mapping (Grad-CAM) technique was used to identify the most important image features for the CNNs.

Earlier, Zhang, *et al.* [13] developed and examined a deep learning system using a CNN or ConvNet for the detection of dental caries using oral photos. The ConvNet algorithm was designed by optimizing the Single Shot MultiBox Detector(SSD) [14] in order to improve cavity detection. Lee *et al.* [15] exploblack the use of deep learning for early detection of dental caries in bitewing radiographs. It highlighted how advanced algorithms improve the accuracy and efficiency of identifying early-stage cavities, potentially enhancing diagnostic capabilities in dental care. In this study, researchers developed a CNN model using a U-shaped deep CNN (U-Net) for caries detection on bitewing radiographs and investigated its impact on improving clinicians' performance. The study complied with ethical regulations. A total of 304 bitewing radiographs were used to train the CNN model, and 50 radiographs were used for performance evaluation.

Several other researchers applied transfer learning or a pre-trained model on lower amounts of data. Prajapati *et al.* [16] and Haghanifar *et al.* [17] experimented with the performance of CNN for diagnosis by employing transfer learning to classify dental caries. Conversely, some methods combined different models for having hybrid model to improve the detection of cavities. Hatvani *et al.* [18] combined two CNN architectures, a subpixel network [19] and U-net [20], to detect cavities. Whereas Zhang *et al.* [21] used Faster R-CNN [22] and R-FCN [23] to detect cavities.

Others utilized different tools and techniques on top of the deep learning model to improve the detection of cavities. Karhade *et al.* [24] developed and evaluated an automated machine learning algorithm (AutoML) [25], using Google Cloud for children's classification according to early childhood caries. Whereas Choi *et al.* in [26] used a preprocessing step (i.e., horizontal alignment of pictublack teeth) followed by a fully convolutional network model with Naïve classifier [27] to detect caries in periapical images. Zanella *et al.* [28] constructed a DenseNet [29] with socioeconomic factors [30] to develop a computer-aided diagnosis tool to detect caries in teeth. Where Moran *et al.* in their early work [31] embedded the image processing tools as a preprocessing step to improve the accuracy of their deep learning model.

The back-propagation in NNs [32], sometime called it (backpropagation NN), shows promising results in classifying the tooth surface as normal or having dental caries [33]. Later, there has been an increase in the number of studies that have used the (U-Net) in the detection and segmentation of caries as proposed by Lee *et al.* [15] and Lian *et al.* [34]. In addition, due to the increasing use of Near-Infrablack Transillumination images (TI) in the detection of caries by dentists, further research has been requiblack in applied deep learning in this type of radiography. Casalegno *et al.* [35] and Schwendicke *et al.* [36] applied CNN with different backbones to detect and localize dental lesions automatically based on TI images. In another study, kuhnisch *et al.* [37] used oral photographs to detect and categorize the cavities. The images were classified as caries-free, non-cavitated caries lesions, or caries-related cavitation. For cyclic training and repeated evaluation of the AI methods, each expert diagnosis served as a reference standard.

Day *et al.* [38] provided a framework to segment dental caries for the Dental Caries Detection Network (DCDNet). DCDNet is characterized by a Multi-Pblackicted Output (MPO) structure at its conclusion. In this regard, it differs from other segmentation architectures. Where, Zhang *et al.* [39] carried out the experiment by combining the adult data with the children's data to demonstrate that, despite significantly increasing the number of children's dental datasets, the adult dataset did not enhance the experimental effects of the children's dental dataset. Earlier this year, Esmaeilyfard *et al.* [40] examined the accuracy of deep learning algorithms in diagnosing tooth caries and classifying the extent and location of dental caries in CBCT images. Currently, this is the first study to evaluate the application of deep learning to CBCT images for detecting dental caries. For identifying both primary and secondary caries lesions, Chaves *et al.* [41] developed a CNN-based automated system using a mask RCNN model. With the support of the Swin Transformer backbone, the study used the Mask-RCNN architecture for segmentation, for instance. The model was trained by ten-fold cross-validation following data augmentation.

Other aim to aid the treatment procedures, Bouchahma *et al.* [42] intended to detect cavities to help the dentist make decisions. They suggested appropriate treatment plans using automatic treatment pblackiction and classification of treatments based on the stage of the cavity.

1.2. Teeth Targeted Studies

1.2.1. Teeth Segmentation

Teeth detection has been a research subject for at least the last two decades, mainly relying on threshold and region-based, and machine learning methods [43]. This paper explores the progress made through machine/deep learning methods in segmenting teeth. The segmentation of teeth from different radiography images has been investigated in sixteen studies.

Jader *et al.*[44] describe the first method of segmenting and recognizing teeth from panoramic X-ray images through a Mask region-based CNN(R-CNN) that employs the addition of a branch for object mask prediction in parallel with the existing branch for class labeling and bounding box recognition [45]. The work of [46] presents a novel 3D dental model segmentation approach via deep CNNs. Later, Tian *et al.* [47] proposed an automated hierarchical teeth segmentation and classification method from 3D dental images based on the sparse voxel OCTREE, conditional random field CRF model, and 3D CNN named O-cnn [48]. Results from the experiment demonstrate high accuracy with potential applications in computer-assisted orthodontic treatment diagnosis. Additionally, Zhu *et al.* [49] developed an automatic teeth detection and segmentation method based on Mask R-CNN. They tested their method on 100 collected images taken from a hospital, and the results demonstrate that the method successfully distinguishes between complex, crowded tooth structures. Additionally, Lee *et al.* in their work [50] attempt to evaluate the performance of a fully(R-CNN) method for automated tooth segmentation using individual annotated panoramic X-ray images.

To avoid ambiguous labeling of other teeth, Sun *et al.* in [51] presented a novel feature steered graph convolutional neural network (FeaStNet) [52] to segment and identify individual teeth from digital dental casts. In order to achieve that, the framework constrains its segmentation and labeling based on the crown shape distribution and concave contours. The proposed method combines the tooth-gingiva and inter-tooth segmentation, rather than carrying them out in separate phases. The accuracy of the proposed method is superior to other DL-based dental segmentation methods: PointNet [53], OCTREE-based CNN [48], and the two-phase cast segmentation methods.

Zhao *et al.* utilized attention networks in [54]. They introduce a Two-Stage Attention Segmentation Network (TSAS-Net) for the tooth localization and segmentation task from publicly available panoramic X-ray images dataset [55]. On the other hand, Cui *et al.* [56] used Generative Adversarial Networks (GAN) [57] to segmenting teeth using comprehensive semantic information. They presented a deep segmentation network based on an

automatic pixel-level tooth segmentation method (ToothPix) based on the conditional-GAN (CGAN) structure. This technique helps to utilize comprehensive semantic information for tooth segmentation from the LNDb dataset [55]. The experimental results showed that the ToothPix method outperformed state-of-the-art methods such as Mask R-CNN and Pix2pix on the LNDb dental dataset. Additionally, Chung *et al.* [58] proposed a CNN for pixel-wise labeling to exploit an instance segmentation framework that is robust to metal artifacts from 3D cone beam computed tomography (CBCT) images.

Zheng *et al.* [59] proposed a novel anatomically constrained dense U-Net [20] for the integration of oral-anatomical knowledge with data-driven dense U-Nets. They aim to enable automated capability for CBCT segmentation and lesion detection.

Additionally, combining different CNN models yields significant results in the segmentation of teeth. Leite *et al.* [60] provide a methodology that aims to assess whether a new AI-based tool is capable of detecting and segmenting teeth from panoramic radiographs. They combined DeepLab-v3 [61] architecture with a pretrained ResNet-101 [62] to detect the teeth and fine-tune the segmentation map of the detector. Chandrashekar *et al.* [63] describes a new technique for integrating tooth segmentation and identification models that were independently created from panoramic radiographs in order to enhance collaborative learning. By using this collaborative method, segmented teeth can be identified and numbered in order to achieve enhanced results.

Fothermore, combining with level set give promising results in segmentation of teeth. Yang *et al.* in their work [64] applied deep CNNs and level sets to segment the teeth from CBCT images. Later, Xie *et al.* [65] in their study, a deep learning method was employed to detect the location and size of each tooth, followed by a method for generating prior ellipses from the boundary boxes detected. Based on the signed distance between each point and the prior edge, the restriction term limits the evolution of level set functions. During the evolution of level set functions, these distances serve as prior weights. A variational model is then applied to separate the joint points of teeth using the curvature direction. Recently,

Rubiu *et al.* [66] in their research, a module was trained utilizing the Mask Region-based Convolutional Neural Network (Mask-RCNN) architecture. A 1000 panoramic dental radiographs from the Tuft dental database served as the source of the training, validation, and testing data. With a Dice index of 0.87, they had a high detection accuracy on the test set (98.4%). Marginean *et al.* [67] proposed a model called CariSeg that trains based on different deep learning models. Using U-Net technology, the area of interest, the teeth, and the radiograph is segmented and cropped around the area of interest. In the next component, carious lesions are segmented using three architectures: U-Net, Feature Pyramid Network, and DeeplabV3.

1.2.2. Tooth Classification

This section contains the tooth classification methods that classify the type of teeth, the problem affecting the teeth, or the condition. Other classification studies focusing on solving other dental fields are distributed in other sections. The classification of tooth types was carried out in seven studies between 2012 and 2023. Where tow study proposed to classified different teeth problems. In addition, there are two other studies that aimed to classified the conditions of teeth.

Imangaliyev *et al.* [68] presents an automated dental red auto-fluorescence plaque image classification model based on the application of CNN to Quantitative Light-induced Fluorescence (QLF) images. Miki *et al.* [69], introduce an application of a deep CNN for classifying tooth types to dental CBCT images. They adapted AlexNet's network architecture [70,71], which consists of five convolution layers, three pooling layers, and two full connection layers, from the Caffe framework [72]. To reduce the effects of overfitting, they applied augmentation techniques to the data including: rotating the images and transforming their intensities. Additionally, Yauney *et al.* [73] reported two CNN classifiers trained with dentist annotations of disease signatures and fluorescent porphyrin biomarker images to identify dental plaque in white light images as a per-pixel binary classification

task. Oktay *et al.* [74] presents a method for detecting teeth in dental panoramic X-ray images with CNN using AlexNet architecture [71] where multi-class classification is performed. Recently, Chen *et al.* [75] classified posterior teeth into eight classes based on (CNN)-based occlusal surface morphology analysis. In order to improve classification performance, image augmentations and deep convolutional generative adversarial networks (DCGANs) [76] were applied to each subnetwork.

Recently, Yilmaz *et al.* [77] applied a comparative analysis of the YOLO-V4 method and the Faster R-CNN method in dental panoramic radiography to determine which is more successful in terms of accuracy, time, and detection ability. Their experiments showed that the YOLO-V4 method was superior to the Faster R-CNN method in terms of accuracy of classification teeth.

Tow study classified teeth problems. Muresan*et al.* [78] presented a novel teeth detection and dental problem classification approach using panoramic dental radiography. The model can highlight 14 different problems that can affect teeth. They applied an Efficient Residual Factorized Convolutional Network (ERFNet) [79] for teeth segmentation and dental problem classification. Almalki *et al.* [80] develop an automated tool that can diagnose and classify dental abnormalities, based on dental panoramic X-ray images, using the YOLOv3 deep learning model.

There are two studies that classified the conditions of teeth. Bacsaran *et al.* [81] evaluated the DL system's ability to identify 10 different dental situations encountered on the panoramic radiographs. The faster R-CNN method [22] and GoogleNet Inception v2 architecture [82] implemented with the TensorFlow library [83] were used for model development.

Muramatsu *et al.* [84] incorporated two tasks (detecting tooth types and conditions) by utilizing a fourfold cross-validation object detection network to classify teeth into four tooth types, including incisors, canines, premolars, and molars, as well as three tooth conditions using a classification network that included nonmetal, restored, partially restored, and completely restored teeth. Vinayahalingam *et al.* [85] in their study, used deep-learning algorithms to assess the accuracy of classifying dental caries on panoramic radiographs. In order to classify carious lesions in mandibular and maxillary third molars based on CNN, MobileNetV2 model was trained on 400 cropped panoramic images.

1.2.3. Detection Of Prostheses and Restorations

Dental Prostheses are dental appliances that a dentist can use to replace or restore a missing tooth or missing parts of tooth structure, or structures that need to be removed to prevent decay. These various prostheses include fillings, crowns and bridges, all of which may cause pain in the future. There have been four studies conducted to detect different types of crowns and dental materials.

Karatas *et al.* in their study [86] examined the success of DL-based CNNs in detecting and differentiating different types of filled teeth: amalgam, composite resin, and metal-ceramic. They applied ResNet-34 architecture [87], a pre-trained module in ImageNet, using two different dental radiography images: bitewing and periapical.

There are also different types of crowns based on the material used to construct them. Takahashi *et al.* [88] develop a method for recognizing dental prostheses and restorations of teeth using deep learning on oral, photographic images. They applied Only Look Once version 3 (YOLOv3) [89] with TensorFlow and Keras libraries to recognize the 11 dental prostheses and restoration types.

Recently, in Panoramic dental images, Imak *et al.* [90] proposed a novel approach to detect dental materials such as fillings, crowns, and bridges. They employed a faster R-CNN to determine the exact location of the tooth region, followed by a Graph cut (GC) [91] to segment the tooth region. The suggested strategy is simple but efficient. Across all classification techniques, the suggested strategy has an average accuracy of about 90%. Oztekin *et al.* [92] developed a deep learning-based method for automatically detecting and classifying amalgam and composite fillings in panoramic images based on U-Nets with

various backbones. Among the various ResNet and ResNext backbones evaluated, the ResNext50 model achieved the highest levels of pixel accuracy and intersection over union (IoU).

1.2.4. Teeth Numbering And Missing Teeth

An important part of a dentist's diagnostic process is the evaluation of dental radiographs. The detection and numbering of teeth is part of the interpretation process carried out by a dental expert. Dental implant placement requires the detection of missing teeth regions. There have been nine studies conducted for teeth numbering and detecting missing teeth.

Raith *et al.* [93] showed that ANNs can be developed to classify dental cusps with sufficient accuracy. In order to generate range image data, 3D surface scans of dental cast representing natural full dental arches were transformed into 3D surface scans. By utilizing an automated algorithm, these data have been processed to identify candidates for tooth cusps based on salient geometrical characteristics. A trained ANN was developed based on the classification of these candidates using common dental terminology. For the first time, this research presents an automated method for classifying teeth that were demonstrated to work with sufficient precision to be applied in clinical practice.

Tuzoff's *et al.* [94] proposed a novel solution based on CNNs to perform panoramic radiographs automatically. Based on the state-of-the-art Faster R-CNN architecture, the model incorporated the classical VGG-16 CNN [95] together with Heuristic Algorithms (HA) [74,96] to improve results according to the rules of tooth arrangement. On the other hand, Chen *et al.* [97] worked on dental periapical films. For detection and numbering of teeth, they developed faster regions with CNN features (faster R-CNN) in the TensorFlow software package. As a complement to the baseline faster R-CNN, they offered three post-processing techniques to improve detection precision based on prior domain knowledge. To eliminate overlapping boxes detected by faster R-CNN associated with the same tooth, a filtering algorithm was first constructed. To detect missing teeth, a neural network model was implemented. Last but not least, a rule-based module based on a tooth numbering system was proposed to modify detected results whose labels violate certain intuitive rules by matching the labels of detected teeth boxes.

Some researchers used combined methods to improve their results. Kim *et al.* [98] presented a new hybrid approach and combined a regional CNN, with a Single Shot Multibox Detector(SSD) and Heuristics Algorithm (HA) [74,96,99]. In this way single teeth and implants can be detected and numbered in a Dental Panoramic X-ray image with only dental fixtures. This approach is extremely useful for providing statistical information and identifying individuals, as well as for separating the images of individual teeth that are used to develop artificial intelligence algorithms based on panoramic X-ray image. Similarly, Lin *et al.* [100] developed a two-phase panoramic X-ray image recognition and classification method by combining data augmentation and preprocessing methods with advanced deep learning techniques to assist dentists in diagnosis. This method began with the automatic classification of tooth position and number from panoramic X-ray tooth images as one of 32 tooth positions. In the second phase, dental conditions were automatically identified from a set of dental conditions specified.

Kilic *et al.* [101] evaluated DL approach to detect and number deciduous teeth on panoramic radiographs of children using an automated approach. In order to detect and number deciduous teeth displayed on pediatric panoramic radiographs, algorithms were developed using Faster R-CNN Inception v2 models [102].

Recently, Park *et al.* [103] proposed a hybrid automated method consisting of two models: segmentation and detection. The method uses panoramic radiographic images to detect regions where teeth are missing. In panoramic radiographic images containing obstacles, such as dental appliances or restorations, segmentation of teeth is necessary in order to detect a missing tooth region accurately. Following the segmentation of teeth, teeth masks were generated based on the segmentation model. In addition, a detection

model was used to determine the areas of missing teeth using the teeth masks as input. In addition, a detection model is used to identify how many and where the missing teeth are located within the panoramic radiographic image. In [104], CNNs were used to detect and classify permanent teeth on orthopantomogram images (OPGs). In order to automate tooth detection and classification, a three-step procedure was developed using CNNs. To begin with, in preliminary segmentation of panoramic images, U-Net performed regions of interest (ROI) detection. Furthermore, each tooth within the ROI determined by the U-Net was identified through the use of the Faster R-CNN. As a third step, the VGG-16 architecture [95] allocated a number to each tooth based on its classification into 32 categories.

Furthermore, Algorithms based on heuristics could improve the accuracy of deep learning algorithms in teeth numbering. karaoglu *et al.* [105] in this study, panoramic dental radiographs were examined to determine the numbering performance of Mask R-CNN and the heuristic algorithm-based method. In the experiment, there was an improvement of more than 4% in the learning-based algorithm. Recently, Ayhan *et al.* [106] based on an improved YOLOv7 model, the study automatically detected and numbered teeth in digital bitewing radiographs obtained from patients. The YOLOv7 algorithm was improved in terms of loss function, backbone network, and activation function. Thus, the model has achieved an improved level of detection accuracy as well as an accelerated rate of detection.

To summarize, Faster R-CNN is the most popular network to improve results in detecting teeth and teeth numbering due to its selective search region proposal generation algorithm.

1.2.5. Detection of Dental Implants

The application of deep learning offers promising performance in computer vision tasks, and is especially suitable for the analysis and recognition of dental images in dental implants [107]. The detection of dental implants has been the subject of eight papers in this systematic review.

Using dental radiographic images collected from three dental hospitals, Lee *et al.* [108] demonstrated the effectiveness of the automated deep CNN, evaluated for the classification of dental implant systems (DISs). A deep CNN automated system demonstrated better accuracy than most of the dental professionals participating in the study. In order to categorize DISs based on dental radiographic images, the automated deep CNN can assist clinical dental practitioners.

Learner *et al.* [109] presented a retrospective clinical study that utilized a full digital protocol utilizing machine learning to produce implant-supported monolithic zirconia crowns (MZCs) cemented on hybrid abutments. A computer-aided design (CAD) program was used to create the individual abutment and temporary crown as part of the study protocol.

When diagnosing and treating patients or responding to complications, appropriate diagnosis and treatment are necessary. Periapical radiographs are essential for correctly classifying implant fixture systems in the absence of detailed medical records. Kim *et al.* [110] investigated whether deep NN and Transfer Learning (TL) modules can recognize four different implant types on intraoral radiographs. In order to determine the optimal pre-trained network architecture, SqueezeNet [111], GoogleNet, ResNet18, MobileNetv2, and ResNet50 were tested. Through deep CNNs and transfer-learning models, Sukegawa *et al.* [99] classified and evaluated the accuracy of identifying different dental implant brands using panoramicX-ray images. An evaluation of five deep CNN models for implant classification was performed (a basic CNN with three convolutional layers, transfer-learning models VGG16 and VGG19 [95], as well as fine-tuned VGG16 and VGG19 models). In terms of classification performance, the VGG16 and VGG19 finely tuned CNNs performed well.

It is essential that dental implant brands and treatment stages are accurately identified in order to ensure efficient care [112]. In this regard, the objective of this study was to

investigate using multi-task deep learning a method for categorizing dental panoramic radiographic images according to implant brands and treatment stages. Five deep CNN models were evaluated, such as ResNet18, 34, 50, 101, and 152. Both classifications were found to perform better with more parameters and a deeper network. A deep NN with two independent outputs was implemented and evaluated as a novel approach in order to determine the brand of implant and the stage of treatment. With CNN's multitasking capabilities, it is possible to analyze both the implant brand and the stage of treatment at the same time.

Kurt *et al.* [113], a three-dimensional Cone-beam Computed Tomography (CBCT) image was used to evaluate the success of the artificial intelligence system in implant planning. An implant planning report is generated by the diagnostic AI system using a pipeline of multiple pre-trained fully NNs (3D U-Net architectures [114]) and an algorithm for extracting slices.

Recently, Park *et al.* [115] in their study used a large-scale multicenter dataset made up of two different types of panoramic and periapical radiography images to assess the precision of an automated deep learning (DL) algorithm for recognizing and classifying different types of dental implant systems (DIS). Using a diverse set of 25 convolutional neural network models, Kurtulus *et al.* [116] conducted a comprehensive analysis. A variety of popular architectures were used in these models, including VGG16, ResNet-50, EfficientNet, and ConvNeXt for panoramic images of X-rays. This study demonstrates the effectiveness of deep learning techniques, particularly the ConvNeXt model, in accurately classifying dental implant systems from panoramic radiographs.

1.2.6. Detection of Bone Loss (Osteoporosis) and Bone Age Measurement (BAM)

In clinical practice, peri-implant bone level detection relies on imaging findings. Commonly used imaging modalities include CBCT, panoramic radiography, and periapical radiography. Furthermore, there are four studies available to estimate the age based on different dental images.

The most common radiographs used in measuring bone loss or age estimation are 343 panoramic and periapical dental radiographs. A total of seven studies used panoramic radiographs. kurt *et al.* [113] aimed in their study to evaluate the use of three-dimensional cone-beam computed tomography (CBCT) images in implant planning using the 3D U-Net model. Both AI and manual measurements of bone thickness had statistically significant differences in all regions of the maxilla and mandible. Later, Lee *et al.* [117] evaluated the diagnostic capabilities of a CNN-based CAD system by comparing them with diagnoses made by oral and maxillofacial radiologists. Additionally, for the purpose of detecting periodontal bone loss (PBL), Krois *et al.* [118] analyzed panoramic dental radiographs with deep CNNs (seven-layer feed-forward CNNs). An image segmentation system based on panoramic radiographs was used to create a set of 2001 image segments. By repeating group shuffles ten times, a deep feed-forward CNN was trained and validated. Grid search was used to tune model architectures and hyperparameters. There were a total of 4,299,651 weights in the final deep NN model that was parameterized by a seven-layer deep NN. A further advance was made by Kim *et al.* [119]. They proposed a deep learning-based approach for the development of an automatic diagnostic system that could detect periodontal bone loss in panoramic dental radiographs. This approach, called DeNTNet, is capable of detecting lesions and providing corresponding teeth numbers according to the notation used by the dental federation.

Avucclu *et al.* [120] proposed a novel algorithm that combined the multiplayer perceptron NNs and image processing techniques to estimate the age of an individual's teeth and bones from panoramic X-ray images. Moreover, in order to stage periodontitis automatically on dental panoramic X-ray radiographs, Chang *et al.* [121] developed a deep learning hybrid method in which deep learning is integrated with machine learning. This paper proposes a novel hybrid framework for the automatic detection and classification of periodontal bone loss among individuals. A method of determining the age of a patient

with panoramic X-ray images was presented by Kahaki *et al.* [122] based on global fuzzy segmentation, local feature extraction, and a deep CNN model designed specifically for this task.

Some other researchers used a periapical instead of a panoramic image to calculate bone loss. The use of periapical radiographs was reported in four studies. Earlier, Huang *et al.* [123] proposed a method to detect the loose bone area from periapical X-ray images. They used a trained weighted average of both the intensity and the texture measured. Later, Cha *et al.* [124] proposed a modified region-based CNN model known as Mask R-CNN. Using the Microsoft Common Objects in Context dataset, the Mask R-CNN was trained using transfer learning. Using periapical radiographic images, this system was proposed in order to determine the percentage of bone loss and classify bone resorption severity. Moran *et al.* [125] evaluated how using resolution improvement methods influences the assessment of periodontal bone loss.

Recently, based on a Faster R-CNN analysis, Liu *et al.* [126] have developed an automated method for determining the degree of bone loss surrounding dental implants in periapical radiographs. Widiasri *et al.* [127] aimed to evaluate how artificial intelligence (AI) can be used to plan dental implants through the use of CBCT images. These images were used to measure bone height and thickness in 508 regions where implants were required. It was also possible to detect canals, sinuses, and fossae associated with alveolar bones and missing tooth regions. A deep convolutional neural network was then used to evaluate all results.

There are four studies available, Sharifonnasabi *et al.* [128] discuss all the details of this newly emerging field. Veleminska *et al.* [129] proposed an accurate predictive ageing system based on RBFNN GAME in order to establish whether there is any difference between the predictive ability of different tooth types during infancy and adolescence and their ontogenetic development stability. They conducted a cross-sectional panoramic X-ray study involving 1393 individuals between the ages of three and seventeen based on a developmental stage assessment of mandibular teeth. Seo *et al.* [130] presented a novel method for estimating bone age based on automatic segmentation of cervical vertebrae from cephalometric projections. The cervical vertebrae were found to provide high accuracy for estimating bone age. As a result of this study, clinicians in the medical and dental fields may consider cephalometric projections as an alternative to hand-wrist radiographs in determining skeletal maturity. Atas *et al.* [131] proposed a modified InceptionV3 model based on deep transfer learning using panoramic X-ray images. According to this study, InceptionV3Mixed 04 has fewer parameters than InceptionV3, thereby delivering faster and more accurate results.

1.2.7. Detection Of Periodontal Diseases

A periodontal disease is an oral inflammation that affects the gingival tissues as well as the tissues supporting the teeth. Aside from the fact that they cause tooth loss, they are also linked to cardiovascular diseases, diabetes, and rheumatoid arthritis. There are six papers for detection of periodontal diseases included in this review.

Lee *et al.* [132] examined the potential usefulness and accuracy of a deep CNN-based computer-aided detection method for identifying and diagnosing periodontally compromised teeth (PCT). In the study, deep CNN architectures were combined with self-trained networks and applied it to periapical radiographic images. On the basis of digital panoramic radiographs, Thanathornwong *et al.* [133] presented a study aimed at identifying periodontally compromised teeth using a deep learning-based object detection method. Based on a small annotated clinical dataset, a faster regional CNN (Faster R-CNN) has been developed.

For detection of gingivitis, using ResNet-50 CNN, two faster region-based CNN models were developed [134]. First, the teeth are detected in order to locate the region of interest (ROI), while secondly, gingival inflammation is detected. Li *et al.*seeks to develop a computer algorithm capable of identifying inflamed disease sites through the use of deep

learning. They have applied their method utilizing the DeepLabv3+ network as well as Xception and MobileNetV2 to the analysis of oral photographs.

For the detection of periodontal pockets, Moriyama *et al.* [135] explored whether deep learning-based imaging (YOLOv2 [136]) would be useful in screening periodontal pockets. They proposed a MapReduce-like method for estimating the depth of periodontal pockets, which overcomes the difficulty of recognizing the pocket from other tissues in oral images.

1.2.8. Detection of Cysts and Tumors

There are six papers for detection of cysts and tumors are included in this review. Okada *et al.* [137] proposed a semi-automatic solution that combined boosted classifiers based on machine learning with graph-based random walks segmentation, and offered a robust clinical test method using CBCT to detect lesions. This study developed a learning model for detecting radiolucent lesions in the mandible using deep NN DetectNets. Ariji *et al.* [138] utilized deep learning to diagnose cervical lymph nodes in patients with oral cancer from computed tomography (CT) images.

Through deep learning (YOLOv3 [89]), Kwon *et al.* [139] was able to automatically diagnose odontogenic cysts and tumors on panoramic radiographs of both jaws. Their work proposes an innovative framework for deep CNNs that allows data augmentation to enhance detection capabilities. Based on the YOLOv3 model, they developed a deep CNN for detecting and classifying tumors of both jaws and odontogenic cysts. Lee *et al.* [140] aimed to determine whether CBCT and X-ray panoramic radiography images using deep neural networks could detect and diagnose three types of odontogenic cystic lesions (OCLs): odontogenic keratocysts, dentigerous cysts, and periapical cysts. An improved detection and diagnosis of OCL was achieved by using GoogleLeNet Inception-v3, which was based on transfer learning. A deep CNN architecture was employed to detect and diagnose odontogenic OCLs in panoramic and CBCT image datasets. Accordingly, CBCT images were found to provide a higher diagnostic performance than panoramic images if a deep CNN architecture was trained with them. An automated model for detecting oral cancer from oral photographs was developed by Warin *et al.* [141] using deep learning algorithms. The classification and detection models were created using DenseNet [142] and faster R-CNN [22], respectively. Where Sivasundaram *et al.* [143] developed an architecture for LeNet [144] using a convolutional neural network with morphology-based segmentation for classifying cyst images and segmenting the cyst regions in each classified cyst image.

In a recent study, Yu *et al.* [145] utilized deep learning to develop a reliable and explainable method to diagnose jaw cysts or tumors based on panoramic radiographs of healthy people. A two-branch network (self supervised sub-branch MoCoV2 [146] and U-net as segmentation sub-branch) was proposed to classify tumors and cysts of the jaw based on 872 lesion samples and 10,000 healthy samples.

1.2.9. Supernumerary and Impacted Wisdom Teeth Detection

"Supernumerary teeth" refer to teeth that are not part of the deciduous or permanent teeth series. Five papers are available for the detection of supernumerary and impacted wisdom teeth. Vinayahalingam *et al.* [147] detected the roots of lower third molars to avoid nerve damage using a DL approach based on U-net. An application of the trained U-net was undertaken to the original panoramic radiographs.

Orhan *et al.* [148] assessed the diagnostic performance of AI based on the deep-CNN system for the evaluation of impacted third molar teeth in CBCT images. The results were evaluated using Kappa analysis statistics [149]. Ha *et al.* [150], on the other hand, developed a model for detecting mesiodens (supernumerary teeth) in panoramic radiographs of different dentition groups using artificial intelligence. A total of 612 panoramic radiographs were used as a training tool. This model for detecting mesiodens was developed based on the YOLOv3 CNN model [89].

Earlier in 2022, Mine *et al.* [151] attempted to detect supernumerary teeth in children by using CNN-based deep learning during the early stages of mixed dentition. A total

of three CNN models were applied to 220 panoramic radiographs taken from children of varying ages: AlexNet, VGG16-TL, and InceptionV3-TL. Among the four models, the VGG16-TL model had the highest accuracy, sensitivity, specificity, and area under the ROC curve. However, the other models also performed well.

1.2.10. Detection of Root (Endodontic) Treatment

There are four papers available regarding the detection of root treatment. Endodontic treatment can be adversely affected by an extra root on the distal root of the mandibular (lower jaw) first molar [152]. A deep learning method was examined by the authors for the purpose of classifying the root anatomy of mandibular first molars on panoramic radiographs based on their diagnostic capabilities. In this study, CBCT was used as the gold standard. A CNN-based deep learning model [153] for detecting vertical root fracture (VRF) on panoramic radiography was built using DetectNet with DIGITS [154]. Five-fold cross-validation was performed to increase the reliability of test data by reducing selection bias.

An apical lesion (AL), was detected by Eker *et al.* [155], on panoramic dental radiographs using a specially designed CNN with seven layers and a total of 4, 299, 651 weights.

In contrast, Mori *et al.* [156] detected the maxillary canine (attached to the upper jaw) based on deep learning models. Their method seeks to evaluate the feasibility and technical quality of maxillary canine positioning using AlexNet [70,71] and U-net [20] for classification and segmentation techniques, respectively.

1.2.11. Detection of Cephalometric Landmark

A growing role has been played by quantitative cephalometry in clinical diagnosis, treatment, and surgery. It is essential to develop fully automated methods for these procedures in order to ensure that computerized analyses are accurate. In this systematic review, five papers discuss the detection of cephalometric landmarks. Earlier, Arik *et al.* [157] proposed an approach using deep convolutional neural networks for the first time to automate quantitative cephalometry. A CNN structure is developed to analyze landmarks that describe patient anatomy and quantify jaw and skull-based pathologies. Qian *et al.* [158] proposed the novel Faster R-CNN-based method, CephaNet, for cephalometric landmark detection. On the basis of the ResNet50 backbone [87], Song *et al.* [159] proposed a two-step method of detecting cephalometric landmarks on skeletal X-ray images. kim *et al.* [160] proposed a CNN based multi-stage landmark identification systemusing combained dataset. Using cone-beam computed tomography (CBCT), the researchers trained and tested multistage CNNs.

Recently, Hong *et al.* [161] applied an automated cephalometric landmark detection using deep Q-networks (DQN) and double deep Q-networks (DDQN) for the first time.Without data augmentation and extra preprocessing, the DQN-based network showed that the average mean radius error of 19 landmarks was less than 2 mm, which is the clinically acceptable threshold.

References

1. Schwendicke, F.; Golla, T.; Dreher, M.; Krois, J. Convolutional neural networks for dental image diagnostics: A scoping review. *Journal of dentistry* **2019**, *91*, 103226.
2. Forouzeshfar, P.; Safaei, A.A.; Ghaderi, F.; Hashemi Kamangar, S.; Kaviani, H.; Haghi, S. Dental caries diagnosis using neural networks and deep learning: a systematic review. *Multimedia Tools and Applications* **2024**, *83*, 30423–30466.
3. Ali, R.B.; Ejbali, R.; Zaied, M. Detection and classification of dental caries in x-ray images using deep neural networks, 2016.
4. Xu, J.; Xiang, L.; Liu, Q.; Gilmore, H.; Wu, J.; Tang, J.; Madabhushi, A. Stacked sparse autoencoder (SSAE) for nuclei detection on breast cancer histopathology images. *IEEE transactions on medical imaging* **2015**, *35*, 119–130.
5. Srivastava, M.M.; Kumar, P.; Pradhan, L.; Varadarajan, S. Detection of tooth caries in bitewing radiographs using deep learning. *arXiv preprint arXiv:1711.07312* **2017**.

6. Long, J.; Shelhamer, E.; Darrell, T. Fully convolutional networks for semantic segmentation. In Proceedings of the Proceedings of the IEEE conference on computer vision and pattern recognition, 2015, pp. 3431–3440.
7. Cantu, A.G.; Gehrung, S.; Krois, J.; Chaurasia, A.; Rossi, J.G.; Gaudin, R.; Elhennawy, K.; Schwendicke, F. Detecting caries lesions of different radiographic extension on bitewings using deep learning. *Journal of Dentistry* **2020**, *100*, 103425.
8. Lee, J.H.; Kim, D.H.; Jeong, S.N.; Choi, S.H. Detection and diagnosis of dental caries using a deep learning-based convolutional neural network algorithm. *Journal of dentistry* **2018**, *77*, 106–111.
9. Sonavane, A.; Yadav, R.; Khamparia, A. Dental cavity classification of using convolutional neural network. In Proceedings of the IOP conference series: materials science and engineering, 2021, Vol. 1022, p. 012116.
10. Bui, T.H.; Hamamoto, K.; Paing, M.P. Deep fusion feature extraction for caries detection on dental panoramic radiographs. *Applied Sciences* **2021**, *11*, 2005.
11. Ding, B.; Zhang, Z.; Liang, Y.; Wang, W.; Hao, S.; Meng, Z.; Guan, L.; Hu, Y.; Guo, B.; Zhao, R.; et al. Detection of dental caries in oral photographs taken by mobile phones based on the YOLOv3 algorithm. *Annals of Translational Medicine* **2021**, *9*.
12. Zheng, L.; Wang, H.; Mei, L.; Chen, Q.; Zhang, Y.; Zhang, H. Artificial intelligence in digital cariology: a new tool for the diagnosis of deep caries and pulpitis using convolutional neural networks. *Annals of Translational Medicine* **2021**, *9*.
13. Zhang, X.; Liang, Y.; Li, W.; Liu, C.; Gu, D.; Sun, W.; Miao, L. Development and evaluation of deep learning for screening dental caries from oral photographs. *Oral Diseases* **2022**, *28*, 173–181.
14. Liu, W.; Anguelov, D.; Erhan, D.; Szegedy, C.; Reed, S.; Fu, C.Y.; Berg, A.C. Ssd: Single shot multibox detector. In Proceedings of the European conference on computer vision, 2016, pp. 21–37.
15. Lee, S.; Oh, S.i.; Jo, J.; Kang, S.; Shin, Y.; Park, J.w. Deep Learning for Early Dental Caries Detection in Bitewing Radiographs, 2021.
16. Prajapati, S.A.; Nagaraj, R.; Mitra, S. Classification of dental diseases using CNN and transfer learning. In Proceedings of the International Symposium on Computational and Business Intelligence (ISCBI), 2017, pp. 70–74.
17. Haghanifar, A.; Majdabadi, M.M.; Ko, S.B. Paxnet: Dental caries detection in panoramic x-ray using ensemble transfer learning and capsule classifier. *arXiv preprint arXiv:2012.13666* **2020**.
18. Hatvani, J.; Horváth, A.; Michetti, J.; Basarab, A.; Kouamé, D.; Gyöngy, M. Deep learning-based super-resolution applied to dental computed tomography. *IEEE Transactions on Radiation and Plasma Medical Sciences* **2018**, *3*, 120–128.
19. Shi, W.; Caballero, J.; Huszár, F.; Totz, J.; Aitken, A.P.; Bishop, R.; Rueckert, D.; Wang, Z. Real-time single image and video super-resolution using an efficient sub-pixel convolutional neural network. In Proceedings of the Proceedings of the IEEE conference on computer vision and pattern recognition, 2016, pp. 1874–1883.
20. Ronneberger, O.; Fischer, P.; Brox, T. U-net: Convolutional networks for biomedical image segmentation. In Proceedings of the International Conference on Medical image computing and computer-assisted intervention. Springer, 2015, pp. 234–241.
21. Zhang, K.; Wu, J.; Chen, H.; Lyu, P. An effective teeth recognition method using label tree with cascade network structure. *Computerized Medical Imaging and Graphics* **2018**, *68*, 61–70.
22. Ren, S.; He, K.; Girshick, R.; Sun, J. Faster r-cnn: Towards real-time object detection with region proposal networks. *Advances in neural information processing systems* **2015**, *28*.
23. Dai, J.; Li, Y.; He, K.; Sun, J. R-fcn: Object detection via region-based fully convolutional networks. *Advances in neural information processing systems* **2016**, *29*.
24. Karhade, D.S.; Roach, J.; Shrestha, P.; Simancas-Pallares, M.A.; Ginnis, J.; Burk, Z.J.; Ribeiro, A.A.; Cho, H.; Wu, D.; Divaris, K. An automated machine learning classifier for early childhood caries. *Pediatric Dentistry* **2021**, *43*, 191–197.
25. LeDell, E.; Poirier, S. H2o automl: Scalable automatic machine learning. In Proceedings of the Proceedings of the AutoML Workshop at ICML, 2020, Vol. 2020.
26. Choi, J.; Eun, H.; Kim, C. Boosting proximal dental caries detection via combination of variational methods and convolutional neural network. *Journal of Signal Processing Systems* **2018**, *90*, 87–97.
27. Rish, I.; et al. An empirical study of the naive Bayes classifier. In Proceedings of the IJCAI 2001 workshop on empirical methods in artificial intelligence, 2001, Vol. 3, pp. 41–46.
28. Zanella-Calzada, L.A.; Galván-Tejada, C.E.; Chávez-Lamas, N.M.; Rivas-Gutierrez, J.; Magallanes-Quintanar, R.; Celaya-Padilla, J.M.; Galván-Tejada, J.I.; Gamboa-Rosales, H. Deep

artificial neural networks for the diagnostic of caries using socioeconomic and nutritional features as determinants: data from NHANES 2013–2014. *Bioengineering* **2018**, *5*, 47.
29. Dongare, A.; Kharde, R.; Kachare, A.D.; et al. Introduction to artificial neural network. *International Journal of Engineering and Innovative Technology (IJEIT)* **2012**, *2*, 189–194.
30. Oakes, J.M.; Rossi, P.H. The measurement of SES in health research: current practice and steps toward a new approach. *Social science & medicine* **2003**, *56*, 769–784.
31. Moran, M.; Faria, M.; Giraldi, G.; Bastos, L.; Oliveira, L.; Conci, A. Classification of Approximal Caries in Bitewing Radiographs Using Convolutional Neural Networks. *Sensors* **2021**, *21*, 5192.
32. Cilimkovic, M. Neural networks and back propagation algorithm. *Institute of Technology Blanchardstown, Blanchardstown Road North Dublin* **2015**, *15*.
33. Geetha, V.; Aprameya, K.; Hinduja, D.M. Dental caries diagnosis in digital radiographs using back-propagation neural network. *Health Information Science and Systems* **2020**, *8*, 1–14.
34. Lian, L.; Zhu, T.; Zhu, F.; Zhu, H. Deep Learning for Caries Detection and Classification. *Diagnostics* **2021**, *11*, 1672.
35. Casalegno, F.; Newton, T.; Daher, R.; Abdelaziz, M.; Lodi-Rizzini, A.; Schürmann, F.; Krejci, I.; Markram, H. Caries detection with near-infrared transillumination using deep learning. *Journal of Dental Research* **2019**, *98*, 1227–1233.
36. Schwendicke, F.; Elhennawy, K.; Paris, S.; Friebertshäuser, P.; Krois, J. Deep learning for caries lesion detection in near-infrared light transillumination images: A pilot study. *Journal of Dentistry* **2020**, *92*, 103260.
37. Kühnisch, J.; Meyer, O.; Hesenius, M.; Hickel, R.; Gruhn, V. Caries detection on intraoral images using artificial intelligence. *Journal of Dental Research* **2022**, *101*, 158–165.
38. Dayı, B.; Üzen, H.; Çiçek, İ.B.; Duman,Ş.B. A Novel Deep Learning-Based Approach for Segmentation of Different Type Caries Lesions on Panoramic Radiographs. *Diagnostics* **2023**, *13*, 202.
39. Zhang, Y.; Ye, F.; Chen, L.; Xu, F.; Chen, X.; Wu, H.; Cao, M.; Li, Y.; Wang, Y.; Huang, X. Children's dental panoramic radiographs dataset for caries segmentation and dental disease detection. *Scientific Data* **2023**, *10*, 380.
40. Esmaeilyfard, R.; Bonyadifard, H.; Paknahad, M. Dental Caries Detection and Classification in CBCT Images Using Deep Learning. *International Dental Journal* **2024**, *74*, 328–334.
41. Chaves, E.T.; Vinayahalingam, S.; van Nistelrooij, N.; Xi, T.; Romero, V.H.D.; Flügge, T.; Saker, H.; Kim, A.; da Silveira Lima, G.; Loomans, B.; et al. Detection of caries around restorations on bitewings using deep learning. *Journal of Dentistry* **2024**, *143*, 104886.
42. Bouchahma, M.; Hammouda, S.B.; Kouki, S.; Alshemaili, M.; Samara, K. An automatic dental decay treatment prediction using a deep convolutional neural network on X-ray images. In Proceedings of the 2019 IEEE/ACS 16th International Conference on Computer Systems and Applications (AICCSA), 2019, pp. 1–4.
43. Chen, X.; Ma, N.; Xu, T.; Xu, C. Deep learning-based tooth segmentation methods in medical imaging: A review. *Proceedings of the Institution of Mechanical Engineers, Part H: Journal of Engineering in Medicine* **2024**, *238*, 115–131.
44. Jader, G.; Fontineli, J.; Ruiz, M.; Abdalla, K.; Pithon, M.; Oliveira, L. Deep instance segmentation of teeth in panoramic X-ray images. In Proceedings of the Conference on Graphics, Patterns and Images (SIBGRAPI), 2018, pp. 400–407.
45. He, K.; Gkioxari, G.; Dollár, P.; Girshick, R. Mask r-cnn. In Proceedings of the Proceedings of the IEEE international conference on computer vision, 2017, pp. 2961–2969.
46. Xu, X.; Liu, C.; Zheng, Y. 3D tooth segmentation and labeling using deep convolutional neural networks. *IEEE transactions on visualization and computer graphics* **2018**, *25*, 2336–2348.
47. Tian, S.; Dai, N.; Zhang, B.; Yuan, F.; Yu, Q.; Cheng, X. Automatic classification and segmentation of teeth on 3D dental model using hierarchical deep learning networks. *IEEE Access* **2019**, *7*, 84817–84828.
48. Wang, P.S.; Liu, Y.; Guo, Y.X.; Sun, C.Y.; Tong, X. O-cnn: Octree-based convolutional neural networks for 3d shape analysis. *ACM Transactions On Graphics (TOG)* **2017**, *36*, 1–11.
49. Zhu, G.; Piao, Z.; Kim, S.C. Tooth detection and segmentation with mask R-CNN. In Proceedings of the 2020 International Conference on Artificial Intelligence in Information and Communication (ICAIIC), 2020, pp. 070–072.
50. Lee, J.H.; Han, S.S.; Kim, Y.H.; Lee, C.; Kim, I. Application of a fully deep convolutional neural network to the automation of tooth segmentation on panoramic radiographs. *Oral Surgery, Oral Medicine, Oral Pathology and Oral Radiology* **2020**, *129*, 635–642.

51. Sun, D.; Pei, Y.; Song, G.; Guo, Y.; Ma, G.; Xu, T.; Zha, H. Tooth segmentation and labeling from digital dental casts. In Proceedings of the 2020 IEEE 17th International Symposium on Biomedical Imaging (ISBI), 2020, pp. 669–673.
52. Verma, N.; Boyer, E.; Verbeek, J. Feastnet: Feature-steered graph convolutions for 3d shape analysis. In Proceedings of the Proceedings of the IEEE conference on computer vision and pattern recognition, 2018, pp. 2598–2606.
53. Qi, C.R.; Su, H.; Mo, K.; Guibas, L.J. Pointnet: Deep learning on point sets for 3d classification and segmentation. In Proceedings of the Proceedings of the IEEE conference on computer vision and pattern recognition, 2017, pp. 652–660.
54. Zhao, Y.; Li, P.; Gao, C.; Liu, Y.; Chen, Q.; Yang, F.; Meng, D. TSASNet: Tooth segmentation on dental panoramic X-ray images by Two-Stage Attention Segmentation Network. *Knowledge-Based Systems* **2020**, *206*, 106338.
55. Silva, G.; Oliveira, L.; Pithon, M. Automatic segmenting teeth in X-ray images: Trends, a novel data set, benchmarking and future perspectives. *Expert Systems with Applications* **2018**, *107*, 15–31.
56. Cui, W.; Zeng, L.; Chong, B.; Zhang, Q. Toothpix: Pixel-Level Tooth Segmentation in Panoramic X-Ray Images based on Generative Adversarial Networks. In Proceedings of the 2021 IEEE 18th International Symposium on Biomedical Imaging (ISBI), 2021, pp. 1346–1350.
57. Liu, M.Y.; Tuzel, O. Coupled generative adversarial networks. *Advances in Neural Information Processing Systems* **2016**, *29*.
58. Chung, M.; Lee, M.; Hong, J.; Park, S.; Lee, J.; Lee, J.; Yang, I.H.; Lee, J.; Shin, Y.G. Pose-aware instance segmentation framework from cone beam CT images for tooth segmentation. *Computers in biology and medicine* **2020**, *120*, 103720.
59. Zheng, Z.; Yan, H.; Setzer, F.C.; Shi, K.J.; Mupparapu, M.; Li, J. Anatomically constrained deep learning for automating dental cbct segmentation and lesion detection. *IEEE Transactions on Automation Science and Engineering* **2020**, *18*, 603–614.
60. Leite, A.F.; Gerven, A.V.; Willems, H.; Beznik, T.; Lahoud, P.; Gaêta-Araujo, H.; Vranckx, M.; Jacobs, R. Artificial intelligence-driven novel tool for tooth detection and segmentation on panoramic radiographs. *Clinical oral investigations* **2021**, *25*, 2257–2267.
61. Chen, L.C.; Papandreou, G.; Schroff, F.; Adam, H. Rethinking atrous convolution for semantic image segmentation. *arXiv preprint arXiv:1706.05587* **2017**.
62. Rao, Y.; He, L.; Zhu, J. A residual convolutional neural network for pan-shaprening. In Proceedings of the 2017 International Workshop on Remote Sensing with Intelligent Processing (RSIP), 2017, pp. 1–4.
63. Chandrashekar, G.; AlQarni, S.; Bumann, E.E.; Lee, Y. Collaborative deep learning model for tooth segmentation and identification using panoramic radiographs. *Computers in Biology and Medicine* **2022**, *148*, 105829.
64. Yang, Y.; Xie, R.; Jia, W.; Chen, Z.; Yang, Y.; Xie, L.; Jiang, B. Accurate and automatic tooth image segmentation model with deep convolutional neural networks and level set method. *Neurocomputing* **2021**, *419*, 108–125.
65. Xie, R.; Yang, Y.; Chen, Z. WITS: Weakly-supervised individual tooth segmentation model trained on box-level labels. *Pattern Recognition* **2023**, *133*, 108974.
66. Rubiu, G.; Bologna, M.; Cellina, M.; Cè, M.; Sala, D.; Pagani, R.; Mattavelli, E.; Fazzini, D.; Ibba, S.; Papa, S.; et al. Teeth Segmentation in Panoramic Dental X-ray Using Mask Regional Convolutional Neural Network. *Applied Sciences* **2023**, *13*, 7947.
67. Mărginean, A.C.; Mureşanu, S.; Hedeşiu, M.; Dioşan, L. Teeth segmentation and carious lesions segmentation in panoramic X-ray images using CariSeg, a networks' ensemble. *Heliyon* **2024**, *10*.
68. Imangaliyev, S.; Veen, M.H.; Volgenant, C.; Keijser, B.J.; Crielaard, W.; Levin, E. Deep learning for classification of dental plaque images. In Proceedings of the International Workshop on Machine Learning, Optimization, and Big Data, 2016, pp. 407–410.
69. Miki, Y.; Muramatsu, C.; Hayashi, T.; Zhou, X.; Hara, T.; Katsumata, A.; Fujita, H. Classification of teeth in cone-beam CT using deep convolutional neural network. *Computers in Biology and Medicine* **2017**, *80*, 24–29.
70. Krizhevsky, A.; Sutskever, I.; Hinton, G.E. Imagenet classification with deep convolutional neural networks. *Advances in neural information processing systems* **2012**, *25*.
71. Krizhevsky, A.; Sutskever, I.; Hinton, G.E. Imagenet classification with deep convolutional neural networks. *Communications of the ACM* **2017**, *60*, 84–90.

72. Jia, Y.; Shelhamer, E.; Donahue, J.; Karayev, S.; Long, J.; Girshick, R.; Guadarrama, S.; Darrell, T. Caffe: Convolutional Architecture for Fast Feature Embedding. *arXiv preprint arXiv:1408.5093* **2014**.
73. Yauney, G.; Angelino, K.; Edlund, D.; Shah, P. Convolutional neural network for combined classification of fluorescent biomarkers and expert annotations using white light images. In Proceedings of the 2017 IEEE 17th International Conference on Bioinformatics and Bioengineering (BIBE), 2017, pp. 303–309.
74. Oktay, A.B. Tooth detection with convolutional neural networks. In Proceedings of the 2017 medical technologies national congress (TIPTEKNO), 2017, pp. 1–4.
75. Chen, Q.; Huang, J.; Salehi, H.S.; Zhu, H.; Lian, L.; Lai, X.; Wei, K. Hierarchical CNN-based occlusal surface morphology analysis for classifying posterior tooth type using augmented images from 3D dental surface models. *Computer Methods and Programs in Biomedicine* **2021**, *208*, 106295.
76. Radford, A.; Metz, L.; Chintala, S. Unsupervised representation learning with deep convolutional generative adversarial networks. *arXiv preprint arXiv:1511.06434* **2015**.
77. Yilmaz, S.; Tasyurek, M.; Amuk, M.; Celik, M.; Canger, E.M. Developing deep learning methods for classification of teeth in dental panoramic radiography. *Oral Surgery, Oral Medicine, Oral Pathology and Oral Radiology* **2023**.
78. Muresan, M.P.; Barbura, A.R.; Nedevschi, S. Teeth Detection and Dental Problem Classification in Panoramic X-Ray Images using Deep Learning and Image Processing Techniques. In Proceedings of the 2020 IEEE 16th International Conference on Intelligent Computer Communication and Processing (ICCP), 2020, pp. 457–463.
79. Romera, E.; Alvarez, J.M.; Bergasa, L.M.; Arroyo, R. Erfnet: Efficient residual factorized convnet for real-time semantic segmentation. *IEEE Transactions on Intelligent Transportation Systems* **2017**, *19*, 263–272.
80. Almalki, Y.E.; Din, A.I.; Ramzan, M.; Irfan, M.; Aamir, K.M.; Almalki, A.; Alotaibi, S.; Alaglan, G.; Alshamrani, H.A.; Rahman, S. Deep Learning Models for Classification of Dental Diseases Using Orthopantomography X-ray OPG Images. *Sensors* **2022**, *22*, 7370.
81. Başaran, M.; Çelik, Ö.; Bayrakdar, I.S.; Bilgir, E.; Orhan, K.; Odabaş, A.; Aslan, A.F.; Jagtap, R. Diagnostic charting of panoramic radiography using deep-learning artificial intelligence system. *Oral Radiology* **2021**, pp. 1–7.
82. Szegedy, C.; Liu, W.; Jia, Y.; Sermanet, P.; Reed, S.; Anguelov, D.; Erhan, D.; Vanhoucke, V.; Rabinovich, A. Going deeper with convolutions. In Proceedings of the Proceedings of the IEEE conference on computer vision and pattern recognition, 2015, pp. 1–9.
83. Abadi, M.; Barham, P.; Chen, J.; Chen, Z.; Davis, A.; Dean, J.; Devin, M.; Ghemawat, S.; Irving, G.; Isard, M.; et al. {TensorFlow}: a system for {Large-Scale} machine learning. In Proceedings of the 12th USENIX symposium on operating systems design and implementation (OSDI 16), 2016, pp. 265–283.
84. Muramatsu, C.; Morishita, T.; Takahashi, R.; Hayashi, T.; Nishiyama, W.; Ariji, Y.; Zhou, X.; Hara, T.; Katsumata, A.; Ariji, E.; et al. Tooth detection and classification on panoramic radiographs for automatic dental chart filing: improved classification by multi-sized input data. *Oral Radiology* **2021**, *37*, 13–19.
85. Vinayahalingam, S.; Kempers, S.; Limon, L.; Deibel, D.; Maal, T.; Hanisch, M.; Bergé, S.; Xi, T. Classification of caries in third molars on panoramic radiographs using deep learning. *Scientific Reports* **2021**, *11*, 1–7.
86. Karatas, O.; Cakir, N.N.; Ozsariyildiz, S.S.; Kis, H.C.; Demirbuga, S.; Gurgan, C.A. A deep learning approach to dental restoration classification from bitewing and periapical radiographs. *Quintessence Int* **2021**, *52*, 568–74.
87. He, K.; Zhang, X.; Ren, S.; Sun, J. Deep Residual Learning for Image Recognition. *CoRR* **2015**, *abs/1512.03385*.
88. Takahashi, T.; Nozaki, K.; Gonda, T.; Mameno, T.; Ikebe, K. Deep learning-based detection of dental prostheses and restorations. *Scientific Reports* **2021**, *11*, 1–7.
89. Redmon, J.; Farhadi, A. Yolov3: An incremental improvement. *arXiv preprint arXiv:1804.02767* **2018**.
90. Imak, A.; Çelebi, A.; Türkoğlu, M.; Şengür, A. Dental Material Detection based on Faster Regional Convolutional Neural Networks and Shape Features. *Neural Processing Letters* **2022**, pp. 1–20.
91. Boykov, Y.; Funka-Lea, G. Graph cuts and efficient ND image segmentation. *International journal of computer vision* **2006**, *70*, 109–131.

92. Oztekin, F.; Katar, O.; Sadak, F.; Aydogan, M.; Yildirim, T.T.; Plawiak, P.; Yildirim, O.; Talo, M.; Karabatak, M. Automatic semantic segmentation for dental restorations in panoramic radiography images using U-Net model. *International Journal of Imaging Systems and Technology* **2022**.
93. Raith, S.; Vogel, E.P.; Anees, N.; Keul, C.; Güth, J.F.; Edelhoff, D.; Fischer, H. Artificial Neural Networks as a powerful numerical tool to classify specific features of a tooth based on 3D scan data. *Computers in biology and medicine* **2017**, *80*, 65–76.
94. Tuzoff, D.V.; Tuzova, L.N.; Bornstein, M.M.; Krasnov, A.S.; Kharchenko, M.A.; Nikolenko, S.I.; Sveshnikov, M.M.; Bednenko, G.B. Tooth detection and numbering in panoramic radiographs using convolutional neural networks. *Dentomaxillofacial Radiology* **2019**, *48*, 20180051.
95. Simonyan, K.; Zisserman, A. Very deep convolutional networks for large-scale image recognition. *arXiv preprint arXiv:1409.1556* **2014**.
96. Eiselt, H.A.; Sandblom, C.L., Heuristic Algorithms. In *Integer Programming and Network Models*; Springer Berlin Heidelberg: Berlin, Heidelberg, 2000; pp. 229–258. https://doi.org/10.1007/978-3-662-04197-0_11.
97. Chen, H.; Zhang, K.; Lyu, P.; Li, H.; Zhang, L.; Wu, J.; Lee, C.H. A deep learning approach to automatic teeth detection and numbering based on object detection in dental periapical films. *Scientific Reports* **2019**, *9*, 1–11.
98. Kim, C.; Kim, D.; Jeong, H.; Yoon, S.J.; Youm, S. Automatic tooth detection and numbering using a combination of a CNN and heuristic algorithm. *Applied Sciences* **2020**, *10*, 5624.
99. Sukegawa, S.; Yoshii, K.; Hara, T.; Yamashita, K.; Nakano, K.; Yamamoto, N.; Nagatsuka, H.; Furuki, Y. Deep neural networks for dental implant system classification. *Biomolecules* **2020**, *10*, 984.
100. Lin, S.Y.; Chang, H.Y. Tooth Numbering and Condition Recognition on Dental Panoramic Radiograph Images Using CNNs. *IEEE Access* **2021**, *9*, 166008–166026.
101. Kılıc, M.C.; Bayrakdar, I.S.; Çelik, Ö.; Bilgir, E.; Orhan, K.; Aydın, O.B.; Kaplan, F.A.; Sağlam, H.; Odabaş, A.; Aslan, A.F.; et al. Artificial intelligence system for automatic deciduous tooth detection and numbering in panoramic radiographs. *Dentomaxillofacial Radiology* **2021**, *50*, 20200172.
102. Szegedy, C.; Vanhoucke, V.; Ioffe, S.; Shlens, J.; Wojna, Z. Rethinking the inception architecture for computer vision. In Proceedings of the Proceedings of the IEEE conference on computer vision and pattern recognition, 2016, pp. 2818–2826.
103. Park, J.; Lee, J.; Moon, S.; Lee, K. Deep Learning Based Detection of Missing Tooth Regions for Dental Implant Planning in Panoramic Radiographic Images. *Applied Sciences* **2022**, *12*, 1595.
104. Estai, M.; Tennant, M.; Gebauer, D.; Brostek, A.; Vignarajan, J.; Mehdizadeh, M.; Saha, S. Deep learning for automated detection and numbering of permanent teeth on panoramic images. *Dentomaxillofacial Radiology* **2022**, *51*, 20210296.
105. Karaoglu, A.; Ozcan, C.; Pekince, A.; Yasa, Y. Numbering teeth in panoramic images: A novel method based on deep learning and heuristic algorithm. *Engineering Science and Technology, an International Journal* **2023**, *37*, 101316.
106. Ayhan, B.; Ayan, E.; Bayraktar, Y. A novel deep learning-based perspective for tooth numbering and caries detection. *Clinical Oral Investigations* **2024**, *28*, 178.
107. Chaurasia, A.; Namachivayam, A.; Koca-Ünsal, R.B.; Lee, J.H. Deep-learning performance in identifying and classifying dental implant systems from dental imaging: a systematic review and meta-analysis. *Journal of Periodontal & Implant Science* **2023**, *54*, 3.
108. Lee, J.H.; Kim, Y.T.; Lee, J.B.; Jeong, S.N. A performance comparison between automated deep learning and dental professionals in classification of dental implant systems from dental imaging: A multi-center study. *Diagnostics* **2020**, *10*, 910.
109. Lerner, H.; Mouhyi, J.; Admakin, O.; Mangano, F. Artificial intelligence in fixed implant prosthodontics: a retrospective study of 106 implant-supported monolithic zirconia crowns inserted in the posterior jaws of 90 patients. *BMC Oral Health* **2020**, *20*, 1–16.
110. Kim, J.E.; Nam, N.E.; Shim, J.S.; Jung, Y.H.; Cho, B.H.; Hwang, J.J. Transfer learning via deep neural networks for implant fixture system classification using periapical radiographs. *Journal of Clinical Medicine* **2020**, *9*, 1117.
111. Iandola, F.N.; Han, S.; Moskewicz, M.W.; Ashraf, K.; Dally, W.J.; Keutzer, K. SqueezeNet: AlexNet-level accuracy with 50x fewer parameters and< 0.5 MB model size. *arXiv preprint arXiv:1602.07360* **2016**.
112. Sukegawa, S.; Yoshii, K.; Hara, T.; Matsuyama, T.; Yamashita, K.; Nakano, K.; Takabatake, K.; Kawai, H.; Nagatsuka, H.; Furuki, Y. Multi-task deep learning model for classification of dental

implant brand and treatment stage using dental panoramic radiograph images. *Biomolecules* **2021**, *11*, 815.
113. Kurt Bayrakdar, S.; Orhan, K.; Bayrakdar, I.S.; Bilgir, E.; Ezhov, M.; Gusarev, M.; Shumilov, E. A deep learning approach for dental implant planning in cone-beam computed tomography images. *BMC Medical Imaging* **2021**, *21*, 1–9.
114. Isensee, F.; Kickingereder, P.; Wick, W.; Bendszus, M.; Maier-Hein, K.H. Brain tumor segmentation and radiomics survival prediction: Contribution to the brats 2017 challenge. In Proceedings of the International MICCAI Brainlesion Workshop, 2017, pp. 287–297.
115. Park, W.; Huh, J.K.; Lee, J.H. Automated deep learning for classification of dental implant radiographs using a large multi-center dataset. *Scientific Reports* **2023**, *13*, 4862.
116. Kurtulus, I.L.; Lubbad, M.; Yilmaz, O.M.D.; Kilic, K.; Karaboga, D.; Basturk, A.; Akay, B.; Nalbantoglu, U.; Yilmaz, S.; Ayata, M.; et al. A robust deep learning model for the classification of dental implant brands. *Journal of Stomatology, Oral and Maxillofacial Surgery* **2024**, p. 101818.
117. Lee, J.S.; Adhikari, S.; Liu, L.; Jeong, H.G.; Kim, H.; Yoon, S.J. Osteoporosis detection in panoramic radiographs using a deep convolutional neural network-based computer-assisted diagnosis system: a preliminary study. *Dentomaxillofacial Radiology* **2019**, *48*, 20170344.
118. Krois, J.; Ekert, T.; Meinhold, L.; Golla, T.; Kharbot, B.; Wittemeier, A.; Dörfer, C.; Schwendicke, F. Deep learning for the radiographic detection of periodontal bone loss. *Scientific Reports* **2019**, *9*, 1–6.
119. Kim, J.; Lee, H.S.; Song, I.S.; Jung, K.H. DeNTNet: Deep Neural Transfer Network for the detection of periodontal bone loss using panoramic dental radiographs. *Scientific Reports* **2019**, *9*, 1–9.
120. Avuçlu, E.; Başçiftçi, F. Novel approaches to determine age and gender from dental X-ray images by using multiplayer perceptron neural networks and image processing techniques. *Chaos, Solitons & Fractals* **2019**, *120*, 127–138.
121. Chang, H.J.; Lee, S.J.; Yong, T.H.; Shin, N.Y.; Jang, B.G.; Kim, J.E.; Huh, K.H.; Lee, S.S.; Heo, M.S.; Choi, S.C.; et al. Deep learning hybrid method to automatically diagnose periodontal bone loss and stage periodontitis. *Scientific Reports* **2020**, *10*, 1–8.
122. Kahaki, S.M.; Nordin, M.; Ahmad, N.S.; Arzoky, M.; Ismail, W.; et al. Deep convolutional neural network designed for age assessment based on orthopantomography data. *Neural Computing and Applications* **2020**, *32*, 9357–9368.
123. Huang, P.W.; Huang, P.Y.; Lin, P.L.; Hsu, H.C. Alveolar bone-loss area detection in periodontitis radiographs using hybrid of intensity and texture analyzed based on FBM model. In Proceedings of the 2014 International Conference on Machine Learning and Cybernetics, 2014, Vol. 2, pp. 487–492.
124. Cha, J.Y.; Yoon, H.I.; Yeo, I.S.; Huh, K.H.; Han, J.S. Peri-implant bone loss measurement using a region-based convolutional neural network on dental periapical radiographs. *Journal of Clinical Medicine* **2021**, *10*, 1009.
125. Moran, M.; Faria, M.; Giraldi, G.; Bastos, L.; Conci, A. Do Radiographic Assessments of Periodontal Bone Loss Improve with Deep Learning Methods for Enhanced Image Resolution? *Sensors* **2021**, *21*, 2013.
126. Liu, M.; Wang, S.; Chen, H.; Liu, Y. A pilot study of a deep learning approach to detect marginal bone loss around implants. *BMC Oral Health* **2022**, *22*, 1–8.
127. Widiasri, M.; Arifin, A.Z.; Suciati, N.; Fatichah, C.; Astuti, E.R.; Indraswari, R.; Putra, R.H.; Za'in, C. Dental-YOLO: Alveolar Bone and Mandibular Canal Detection on Cone Beam Computed Tomography Images for Dental Implant Planning. *IEEE Access* **2022**, *10*, 101483–101494.
128. Sharifonnasabi, F.; Jhanjhi, N.; John, J.; Alaboudi, A.; Nambiar, P. A Review on Automated Bone Age Measurement Based on Dental OPG Images. *International Journal of Engineering Research and Technology* **2020**, *13*, 5408–5422.
129. Velemínská, J.; Pílnỳ, A.; Cepek, M.; Kot'ová, M.; Kubelková, R. Dental age estimation and different predictive ability of various tooth types in the Czech population: data mining methods. *Anthropologischer Anzeiger; Bericht Uber die Biologisch-anthropologische Literatur* **2013**, *70*, 331–345.
130. Seo, H.; Hwang, J.; Jung, Y.H.; Lee, E.; Nam, O.H.; Shin, J. Deep focus approach for accurate bone age estimation from lateral cephalogram. *Journal of Dental Sciences* **2022**.
131. Atas, I.; Ozdemir, C.; Atas, M.; Dogan, Y. Forensic Dental Age Estimation Using Modified Deep Learning Neural Network. *arXiv preprint arXiv:2208.09799* **2022**.
132. Lee, J.H.; Kim, D.h.; Jeong, S.N.; Choi, S.H. Diagnosis and prediction of periodontally compromised teeth using a deep learning-based convolutional neural network algorithm. *Journal of Periodontal & Implant Science* **2018**, *48*, 114–123.

133. Thanathornwong, B.; Suebnukarn, S. Automatic detection of periodontal compromised teeth in digital panoramic radiographs using faster regional convolutional neural networks. *Imaging Science in Dentistry* **2020**, *50*, 169.
134. Alalharith, D.M.; Alharthi, H.M.; Alghamdi, W.M.; Alsenbel, Y.M.; Aslam, N.; Khan, I.U.; Shahin, S.Y.; Dianišková, S.; Alhareky, M.S.; Barouch, K.K. A deep learning-based approach for the detection of early signs of gingivitis in orthodontic patients using faster region-based convolutional neural networks. *International Journal of Environmental Research and Public Health* **2020**, *17*, 8447.
135. Moriyama, Y.; Lee, C.; Date, S.; Kashiwagi, Y.; Narukawa, Y.; Nozaki, K.; Murakami, S. A MapReduce-like Deep Learning Model for the Depth Estimation of Periodontal Pockets. In Proceedings of the HEALTHINF, 2019, pp. 388–395.
136. Redmon, J.; Farhadi, A. YOLO9000: better, faster, stronger. In Proceedings of the Proceedings of the IEEE conference on computer vision and pattern recognition, 2017, pp. 7263–7271.
137. Okada, K.; Rysavy, S.; Flores, A.; Linguraru, M.G. Noninvasive differential diagnosis of dental periapical lesions in cone-beam CT scans. *Medical Physics* **2015**, *42*, 1653–1665.
138. Ariji, Y.; Fukuda, M.; Kise, Y.; Nozawa, M.; Yanashita, Y.; Fujita, H.; Katsumata, A.; Ariji, E. Contrast-enhanced computed tomography image assessment of cervical lymph node metastasis in patients with oral cancer by using a deep learning system of artificial intelligence. *Oral Surgery, Oral Medicine, Oral Pathology and Oral Radiology* **2019**, *127*, 458–463.
139. Kwon, O.; Yong, T.H.; Kang, S.R.; Kim, J.E.; Huh, K.H.; Heo, M.S.; Lee, S.S.; Choi, S.C.; Yi, W.J. Automatic diagnosis for cysts and tumors of both jaws on panoramic radiographs using a deep convolution neural network. *Dentomaxillofacial Radiology* **2020**, *49*, 20200185.
140. Lee, J.H.; Kim, D.H.; Jeong, S.N. Diagnosis of cystic lesions using panoramic and cone beam computed tomographic images based on deep learning neural network. *Oral diseases* **2020**, *26*, 152–158.
141. Warin, K.; Limprasert, W.; Suebnukarn, S.; Jinaporntham, S.; Jantana, P. Automatic classification and detection of oral cancer in photographic images using deep learning algorithms. *Journal of Oral Pathology & Medicine* **2021**, *50*, 911–918.
142. Huang, G.; Liu, Z.; Van Der Maaten, L.; Weinberger, K.Q. Densely connected convolutional networks. In Proceedings of the Proceedings of the IEEE conference on computer vision and pattern recognition, 2017, pp. 4700–4708.
143. Sivasundaram, S.; Pandian, C. Performance analysis of classification and segmentation of cysts in panoramic dental images using convolutional neural network architecture. *International Journal of Imaging Systems and Technology* **2021**, *31*, 2214–2225.
144. LeCun, Y.; Bottou, L.; Bengio, Y.; Haffner, P. Gradient-based learning applied to document recognition. *Proceedings of the IEEE* **1998**, *86*, 2278–2324.
145. Yu, D.; Hu, J.; Feng, Z.; Song, M.; Zhu, H. Deep learning based diagnosis for cysts and tumors of jaw with massive healthy samples. *Scientific Reports* **2022**, *12*, 1–10.
146. Chen, X.; Fan, H.; Girshick, R.; He, K. Improved baselines with momentum contrastive learning. *arXiv preprint arXiv:2003.04297* **2020**.
147. Vinayahalingam, S.; Xi, T.; Bergé, S.; Maal, T.; de Jong, G. Automated detection of third molars and mandibular nerve by deep learning. *Scientific reports* **2019**, *9*, 1–7.
148. Orhan, K.; Bilgir, E.; Bayrakdar, I.S.; Ezhov, M.; Gusarev, M.; Shumilov, E. Evaluation of artificial intelligence for detecting impacted third molars on cone-beam computed tomography scans. *Journal of Stomatology, Oral and Maxillofacial Surgery* **2021**, *122*, 333–337.
149. Donner, A.; Klar, N. The statistical analysis of kappa statistics in multiple samples. *Journal of clinical epidemiology* **1996**, *49*, 1053–1058.
150. Ha, E.G.; Jeon, K.J.; Kim, Y.H.; Kim, J.Y.; Han, S.S. Automatic detection of mesiodens on panoramic radiographs using artificial intelligence. *Scientific reports* **2021**, *11*, 1–8.
151. Mine, Y.; Iwamoto, Y.; Okazaki, S.; Nakamura, K.; Takeda, S.; Peng, T.Y.; Mitsuhata, C.; Kakimoto, N.; Kozai, K.; Murayama, T. Detecting the presence of supernumerary teeth during the early mixed dentition stage using deep learning algorithms: A pilot study. *International Journal of Paediatric Dentistry* **2022**, *32*, 678–685.
152. Hiraiwa, T.; Ariji, Y.; Fukuda, M.; Kise, Y.; Nakata, K.; Katsumata, A.; Fujita, H.; Ariji, E. A deep-learning artificial intelligence system for assessment of root morphology of the mandibular first molar on panoramic radiography. *Dentomaxillofacial Radiology* **2019**, *48*, 20180218.
153. Fukuda, M.; Inamoto, K.; Shibata, N.; Ariji, Y.; Yanashita, Y.; Kutsuna, S.; Nakata, K.; Katsumata, A.; Fujita, H.; Ariji, E. Evaluation of an artificial intelligence system for detecting vertical root fracture on panoramic radiography. *Oral Radiology* **2020**, *36*, 337–343.

154. Ariji, Y.; Yanashita, Y.; Kutsuna, S.; Muramatsu, C.; Fukuda, M.; Kise, Y.; Nozawa, M.; Kuwada, C.; Fujita, H.; Katsumata, A.; et al. Automatic detection and classification of radiolucent lesions in the mandible on panoramic radiographs using a deep learning object detection technique. *Oral Surgery, Oral Medicine, Oral Pathology and Oral Radiology* **2019**, *128*, 424–430.
155. Ekert, T.; Krois, J.; Meinhold, L.; Elhennawy, K.; Emara, R.; Golla, T.; Schwendicke, F. Deep learning for the radiographic detection of apical lesions. *Journal of endodontics* **2019**, *45*, 917–922.
156. Mori, M.; Ariji, Y.; Fukuda, M.; Kitano, T.; Funakoshi, T.; Nishiyama, W.; Kohinata, K.; Iida, Y.; Ariji, E.; Katsumata, A. Performance of deep learning technology for evaluation of positioning quality in periapical radiography of the maxillary canine. *Oral Radiology* **2022**, *38*, 147–154.
157. Arik, S.Ö.; Ibragimov, B.; Xing, L. Fully automated quantitative cephalometry using convolutional neural networks. *Journal of Medical Imaging* **2017**, *4*, 014501.
158. Qian, J.; Cheng, M.; Tao, Y.; Lin, J.; Lin, H. CephaNet: An improved faster R-CNN for cephalometric landmark detection. In Proceedings of the 2019 IEEE 16th International Symposium on Biomedical Imaging (ISBI 2019), 2019, pp. 868–871.
159. Song, Y.; Qiao, X.; Iwamoto, Y.; Chen, Y.w. Automatic cephalometric landmark detection on X-ray images using a deep-learning method. *Applied Sciences* **2020**, *10*, 2547.
160. Kim, M.J.; Liu, Y.; Oh, S.H.; Ahn, H.W.; Kim, S.H.; Nelson, G. Automatic cephalometric landmark identification system based on the multi-stage convolutional neural networks with CBCT combination images. *Sensors* **2021**, *21*, 505.
161. Hong, W.; Kim, S.M.; Choi, J.; Ahn, J.; Paeng, J.Y.; Kim, H. Automated Cephalometric Landmark Detection Using Deep Reinforcement Learning. *Journal of Craniofacial Surgery* **2023**, pp. 10–1097.
